# Multi-region brain transcriptomic analysis of amyotrophic lateral sclerosis reveals widespread RNA alterations and substantial cerebellum involvement

**DOI:** 10.1186/s13024-025-00820-5

**Published:** 2025-04-25

**Authors:** Natalie Grima, Andrew N. Smith, Claire E. Shepherd, Lyndal Henden, Thiri Zaw, Luke Carroll, Dominic B. Rowe, Matthew C. Kiernan, Ian P. Blair, Kelly L. Williams

**Affiliations:** 1 Macquarie University Motor Neuron Disease Research Centre, Faculty of Medicine, Health and Human Sciences, Macquarie University, Sydney, NSW 2109 Australia; 2 Neuroscience Research Australia, Randwick, NSW 2031 Australia; 3 Australian Proteome Research Facility, Macquarie University, Sydney, NSW 2109 Australia; 4 Brain and Mind Centre, The University of Sydney, Sydney, NSW 2050 Australia; 5 Department of Neurology, Royal Prince Alfred Hospital, Sydney, NSW 2050 Australia

**Keywords:** Amyotrophic lateral sclerosis, TDP-43 pathology, RNA-seq, Transcriptome, Alternative splicing, Cryptic splicing, Cerebellum, Post-mortem

## Abstract

**Background:**

Amyotrophic lateral sclerosis (ALS) is a neurodegenerative disease that primarily affects the motor neurons, causing progressive muscle weakness and paralysis. While research has focused on understanding pathological mechanisms in the motor cortex and spinal cord, there is growing evidence that extra-motor brain regions may also play a role in the pathogenesis or progression of ALS.

**Methods:**

We generated 165 sample-matched post-mortem brain transcriptomes from 22 sporadic ALS patients with pTDP-43 pathological staging and 11 non-neurological controls. For each individual, five brain regions underwent mRNA sequencing: motor cortex (pTDP-43 inclusions always present), prefrontal cortex and hippocampus (pTDP-43 inclusions sometimes present), and occipital cortex and cerebellum (pTDP-43 inclusions rarely present). We examined gene expression, cell-type composition, transcript usage (% contribution of a transcript to total gene expression) and alternative splicing, comparing ALS-specific changes between brain regions. We also considered whether post-mortem pTDP-43 pathological stage classification defined ALS subgroups with distinct gene expression profiles.

**Results:**

Significant gene expression changes were observed in ALS cases for all five brain regions, with the cerebellum demonstrating the largest number of total (> 3,000) and unique (60%) differentially expressed genes. Pathway enrichment and predicted activity were largely concordant across brain regions, suggesting that ALS-linked mechanisms, including inflammation, mitochondrial dysfunction and oxidative stress, are also dysregulated in non-motor brain regions. Switches in transcript usage were identified for a small set of genes including increased usage of a *POLDIP3* transcript, associated with TDP-43 loss-of-function, in the cerebellum and a *XBP1* transcript, indicative of unfolded protein response activity, in the motor cortex. Extensive variation in RNA splicing was identified in the ALS brain, with 26–41% of alternatively spliced genes unique to a given brain region. This included detection of TDP-43-associated cryptic splicing events such as the *STMN2* cryptic exon which was shown to have a pTDP-43 pathology-specific expression pattern. Finally, ALS patients with stage 4 pTDP-43 pathology demonstrated distinct gene and protein expression changes in the cerebellum.

**Conclusions:**

Together our findings highlighted widespread transcriptome alterations in ALS post-mortem brain and showed that, despite the absence of pTDP-43 pathology in the cerebellum, extensive and pTDP-43 pathological stage-specific RNA changes are evident in this brain region.

**Supplementary Information:**

The online version contains supplementary material available at 10.1186/s13024-025-00820-5.

## Background

In amyotrophic lateral sclerosis (ALS), degeneration of the motor neurons present in the motor cortex, brainstem and spinal cord results in the progressive loss of voluntary motor control. While these specific central nervous system (CNS) regions have consequently garnered the greatest research interest, there is increasing evidence that pathological changes in ALS patients are not confined to motor regions. For one, ALS is considered to exist on a disease spectrum with frontotemporal dementia (FTD), a neurodegenerative disorder affecting the frontal and temporal lobes of the brain. Cognitive deficits are reported in more than half of all ALS patients, with around 15% diagnosed with comorbid FTD [1]. Overlapping genetic causes [2] and neuropathological substrate [3] further support the existence of common pathomechanisms leading to neurodegeneration in ALS and FTD. Until recently, there has been little focus on the cerebellum in ALS, which plays a role in motor coordination. Rationale for examination of the cerebellum in ALS was bolstered following the identification of repeat expansions in two genes, *C9orf72* and *ATXN2*, that confer ALS risk and cause cerebellar pathologies [4–6]. Pathogenic repeat expansions in *C9orf72* are present in approximately 10% of all ALS patients and cause the formation of dipeptide-repeat protein inclusions in the cerebellum [7]. For *ATXN2*, intermediate expansions (29–33 repeats) are associated with ALS risk while expansions > 33 repeats cause spinocerebellar ataxia type 2 (SCA2), a neurodegenerative disease which primarily affects cerebellar Purkinje neurons [6]. Neuroimaging studies have also played a prominent role in implicating various non-motor regions by identifying broader structural or metabolic changes in the CNS of ALS patients [8]. However, mapping a neuroimaging phenotype back to a neuropathological and further still, molecular alteration, is complex. For example, while neuroimaging studies have identified cerebellar atrophy in ALS patients with various genetic backgrounds [9, 10], histopathological assessment has suggested that cerebellar neuronal loss only occurs in ALS patients with intermediate repeat expansions in *ATXN2* [11]. Further molecular-level investigation is therefore required to more precisely characterise the changes that take place in ALS patient brains. This will also be critical to understanding whether extra-motor regions play a role in the pathogenesis or progression of ALS.

**Fig. 1:**
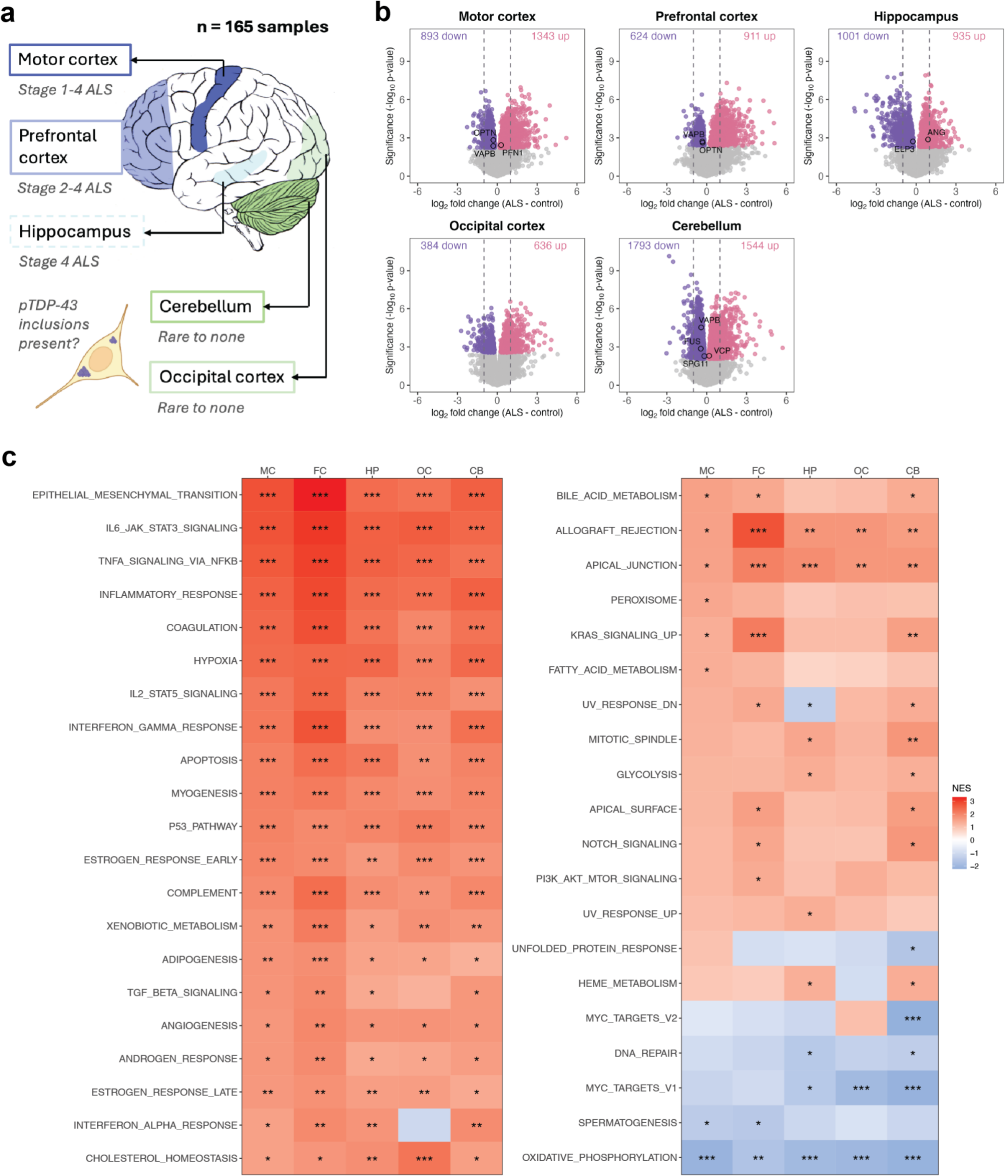
Widespread gene expression changes in ALS post-mortem brain. **(a)** The five brain regions examined by RNA sequencing for 22 sporadic ALS patients and 11 controls (*n* = 165 samples). According to the staging scheme, motor cortex presents with pTDP-43 inclusions in stage 1, 2, 3 and 4 ALS patients, prefrontal cortex presents with pTDP-43 inclusions in stage 2, 3 and 4 ALS patients, hippocampus presents with pTDP-43 inclusions in stage 4 ALS patients, and cerebellum and occipital cortex rarely present with pTDP-43 pathology. **(b)** Volcano plots showing the genes that were significantly upregulated (in red) or downregulated (in blue) in the ALS patients relative to controls (FDR < 0.05). Dashed lines indicate the boundary for a 2-fold increase and decrease in gene expression. Genes that have been genetically implicated in ALS, and which were significantly differentially expressed, are labelled. **(c)** Gene set enrichment analysis result for the MSigDB human hallmark gene sets. Tiles are coloured by the normalised-enrichment score (NES) indicating whether a particular gene set was enriched or depleted in ALS patients relative to controls. P-values are adjusted using the Benjamini Hochberg method (*, adjusted p-value < 0.05; **, adjusted p-value < 10^− 3^; ***, adjusted p-value < 10^− 5^). MC, motor cortex; FC, prefrontal cortex; HP, hippocampus; OC, occipital cortex; CB, cerebellum

Intraneuronal aggregates of phosphorylated TAR DNA-binding protein (pTDP-43) are a hallmark feature of ALS, present in around 97% of all patients [3]. Pathological TDP-43 has been observed in multiple brain regions beyond the motor cortex supporting the proposition that ALS is a multisystem neurodegenerative disorder [12]. However, the incidence of this pathology throughout the CNS is heterogeneous, occurring in different neuroanatomical regions among patients. Four neuropathological stages, defined by the post-mortem location of pTDP-43 inclusion pathology, have been identified [13, 14] (Fig. 1a). In stage 1 ALS patients, pTDP-43 pathology is present in the agranular motor cortex, in spinal cord α-motor neurons and brainstem motor nuclei of select cranial nerves. Stage 2 ALS patients display pTDP-43 pathology in stage 1 regions plus prefrontal neocortex, reticular formation, precerebellar nuclei and red nucleus, while stage 3 ALS patients are defined by further pathology in the prefrontal and postcentral neocortex and striatum. Stage 4 ALS patients have the most extensive regional burden of pTDP-43 pathology, with pathology identified in the anteromedial temporal lobe, including the hippocampus, in addition to stage 1–3 regions. The accumulating number of CNS regions impacted by pTDP-43 pathology suggests a pathological spread from regions that always display pathology in ALS (i.e. stage 1 regions) to those that less frequently display pathology (i.e. stage 4 regions). It is unclear what underlies this region-specific susceptibility to pTDP-43 pathology and whether the consequence of its presence is variable across the CNS. Furthermore, while there have been some limited clinical associations with this post-mortem staging scheme [14], it is not known whether it defines biologically distinct groups beyond the incidence of pTDP-43 pathology.

In this study, we aimed to explore molecular alterations that occur in different regions of ALS patient brain by RNA sequencing (RNA-seq) of post-mortem tissue. To date, several CNS regions have been examined by bulk RNA-seq of ALS post-mortem tissue including motor cortex [15, 16], frontal cortex [15, 17, 18], cerebellum [17, 19, 20] and spinal cord [21–23]. While these studies have highlighted numerous divergent biological pathways in ALS, only Prudencio et al. [17] examined more than one CNS region (frontal cortex and cerebellum) from the same set of individuals. The use of independent ALS patient cohorts between studies, has limited direct comparisons between different brain regions. Another important consideration is the verification of pTDP-43 pathology in post-mortem samples, particularly in brain regions known to variably present with pTDP-43 pathology in ALS. This is critical for transcriptomic studies, given the established role of TDP-43 in RNA metabolism, including the association of TDP-43 pathology with the inclusion of cryptic exons in neuronally expressed genes such as *STMN2* [24, 25] and *UNC13A* [26, 27]. To address these considerations, we performed RNA-seq on post-mortem brain derived from 22 sporadic ALS patients with pTDP-43 pathological staging and 11 non-neurological controls. For each individual, we examined five brain regions that are differentially impacted by pTDP-43 pathology (*n* = 165 matched samples). We performed a comprehensive analysis of ALS-control differences per brain region including differential gene expression, cell-type deconvolution, differential transcript usage and alternative splicing analyses. We also considered whether the pTDP-43 pathological stage classification defined ALS subgroups with distinct gene expression profiles. Together our findings highlighted widespread transcriptome alterations in ALS post-mortem brain and suggested that, despite the absence of pTDP-43 pathology in the cerebellum, changes in the cerebellum may occur in response to regional spread of pTDP-43 pathology elsewhere in the brain.

## Methods

### Study cohort

Post-mortem fresh-frozen brain tissue was obtained for 22 sporadic ALS cases and 11 neurologically healthy control participants from Sydney Brain Bank (Neuroscience Research Australia) and the New South Wales Brain Tissue Resource Centre (The University of Sydney, Australia) (Table 1). ALS cases were clinically diagnosed according to El Escorial criteria [28] and were classified as sporadic based on the absence of a family history of neurodegenerative disease. ALS patients did not carry a pathogenic repeat expansion in *C9orf72* (defined as > 30 repeats) as confirmed by repeat-primed PCR and fragment analysis (method previously described [5]). No ALS patients had intermediate (29–33) or pathogenic (≥34) repeat expansions in *ATXN2* as confirmed by PCR and fragment analysis. ALS patients were also confirmed to not carry a pathogenic variant in *SOD1* by PCR and Sanger sequencing. For each individual, post-mortem tissue was collected as previously described [29] from five brain regions: motor cortex, prefrontal cortex, occipital cortex, hippocampus and cerebellum (*n* = 165 tissue samples). De-identified demographic and clinical characteristics of ALS cases and control participants are presented in Table S1.

**Table 1:** Demographic and clinical information for the study cohort

	Control	ALS			*p* value	
		Stage 1	Stage 2–3	Stage 4	ALS vs. Control	Inter-stage
Donors	11	6	9 (2 stage 3)	7	-	-
Sex (% males)	6 (54.5%)	5 (83.3%)	6 (66.7%)	2 (28.6%)	1	0.141
Age at death (years)	78.1 ± 11.1	62.7 ± 12.8	62.7 ± 13.5	71.1 ± 3.7	**0.006**	0.274
Post-mortem delay (hours)	26.8 ± 13.6	29.3 ± 18.7	23.3 ± 17.7	20.1 ± 13.8	0.607	0.621
Cerebellar tissue pH	6.6 ± 0.3	6.1 ± 0.2	6.3 ± 0.4	6.3 ± 0.4	**0.005**	0.622
Age at onset (years)	-	59.4 ± 13.6	59.4 ± 12.3	67.2 ± 5.1	-	0.319
Disease duration (months)	-	42.2 ± 17.1	40.4 ± 16.2	46.0 ± 12.2	-	0.913
Site of onset	-				-	0.703
Bulbar	-	2 (33.3%)	3 (33.4%)	1 (14.3%)	-	-
Limb	-	3 (50%)	6 (66.7%)	5 (71.4%)	-	-
Truncal	-	1 (16.7%)	0	1 (14.3%)	-	-

Age at death, post-mortem delay, cerebellar tissue pH, age at onset and disease duration are presented as mean ± standard deviation. ALS vs. control comparisons were performed using a Pearson’s Chi-squared test for categorical variables and a Welch two sample t-test for continuous variables. Inter-stage comparisons (i.e. stage 1 ALS vs. stage 2–3 ALS vs. stage 4 ALS) was performed using a Fisher’s exact test for categorical variables, a one-way ANOVA for continuous variables and a Kaplan-Meier estimate for disease duration. Presented p values are uncorrected for multiple testing with significant values (*p* < 0.05) shown in bold. Statistical results for all biological and technical metrics are provided in Tables S4 and S5

### pTDP-43 pathological staging

ALS patients underwent post-mortem pTDP-43 pathological staging [13] by an experienced neuropathologist. The severity of pTDP-43 inclusion pathology was semi-quantitatively graded in each region as none, mild, moderate or severe. ALS patients were classified as stage 1 (*n* = 6), stage 2 (*n* = 7), stage 3 (*n* = 2) or stage 4 (*n* = 7) pTDP-43 pathology. Stage 2 and stage 3 ALS patients were grouped together for all analyses due to the small number of stage 3 cases and the absence of a brain region distinguishing the two pathology stages in the present study.

### RNA extraction and sequencing

RNA was isolated from fresh-frozen post-mortem brain tissue using the AllPrep DNA/RNA mini kit (Qiagen, Hilden, Germany) as previously described [29]. RNA integrity was measured using the Agilent RNA 6000 Nano assay on the Agilent 2100 Bioanalyzer system (Agilent Technologies, Santa Clara, CA). All sequenced samples had an RNA integrity number (RIN) ≥ 6. RIN was also measured by the third-party sequencing provider using the Agilent TapeStation system (Agilent Technologies). RNA-seq libraries were prepared from total RNA using the TruSeq Stranded mRNA Library Prep Kit (Illumina, CA, USA). Sequencing was performed on an Illumina NovaSeq 6000 platform (151 bp paired-end reads) generating raw sequencing reads in FASTQ format (Macrogen, South Korea).

### RNA-seq data processing

An overview of the RNA-seq data processing and analysis pipeline is presented in Figure S1. Raw FASTQ files were processed using Trimmomatic v0.38 to remove low-quality reads and adaptor sequences [30]. Trimmed FASTQ files were then aligned to the GENCODE 43 human reference genome (GRCh38.primary_assembly) using STAR v2.7.10b with–twopassMode Basic parameter specified [31]. Gene counts were generated using RSEM v1.3.3 [32] and were imported into R using tximport v1.28.0 [33]. Quality check was performed using FastQC v0.11.9 (http://www.bioinformatics.babraham.ac.uk/projects/fastqc/), MultiQC v1.12 [34] and Picard CollectRnaSeqMetrics tool (http://broadinstitute.github.io/picard). Per sample technical characteristics and sequencing metrics are presented in Table S2. All following analyses were performed in R [35] unless specified otherwise. Following filtering of low count genes (< 10 counts in < 11 samples), gene counts were normalised and log transformed using a variance stabilizing transformation [36]. Principal component analysis (PCA) was performed on the resulting expression matrix using prcomp and samples were visualised by 2D plotting of principal components (PCs) to check for outliers. The same analysis was performed on brain RNA-seq data available from the Genotype Tissue Expression (GTEx) Consortium to visualise the expected distribution of brain regions (frontal_cortex_ba9, cerebellar_hemisphere and brain_hippocampus; 2017-06-05_v8; *n* = 621 samples).

### Confounder identification and adjustment

Confounder identification and adjustment was performed separately for each brain region. First, biological and technical variables were visualised by 2D plotting of PCs to identify any obvious associations between reported variables and gene expression. All variables were then ranked based on their minimisation of the Bayesian Information Criterion (BIC) as determined using the limma selectModel function [37] (Table S3). The number of genes with a lower BIC in the tested model was used to indicate whether a given variable improved the base model containing only disease status as a predictor. To avoid multicollinearity, correlated variables with a Pearson correlation co-efficient ≥ 0.5 (covariates only) or Canonical Correlation Analysis co-efficient ≥ 0.5 were identified and only the variable with the highest BIC rank was retained. To reduce the remaining independent variables to those with a substantial influence on gene expression variance, two further filtering steps were performed. First, the five variables with the greatest contribution to total variance were identified using variancePartition [38] (Figure S2). A differential gene expression analysis was then performed using limma voom [37] using the model “∼ disease status + sex + age + 5 top ranked variables”. Variables with ≥ 10% genes called as differentially expressed (FDR < 0.05) were included in the final model. The linear models fitted for each brain region are listed in Table 2.

**Table 2:** Linear models employed for ALS-control analyses

Brain region	Model
Motor cortex	∼ % multi-mapped reads + GC content + age + sex + disease status
Prefrontal cortex	∼ % multi-mapped reads + % mRNA bases + GC content + age + sex + disease status
Hippocampus	∼ % UTR bases + % intergenic bases + age + sex + disease status
Occipital cortex	∼ % UTR bases + age + sex + disease status
Cerebellum	∼ TapeStation RIN + GC content + age + sex + disease status

### Differential gene expression analysis

Following filtering of low-count genes using edgeR v3.42.4 filterByExpr function [39], differential gene expression analysis was performed using limma v3.56.2 voomWithQualityWeights function [37, 40]. Genes with a Benjamini–Hochberg adjusted p-value (FDR) < 0.05 were considered differentially expressed. Two differential gene expression analyses were performed. First, differential gene expression analysis was performed between ALS patients and controls for each brain region adjusting for identified confounders (Table 2). Log_2_ fold changes of each gene were correlated between each pair of brain regions to assess the similarity of changes between brain regions. A second comparison was performed between ALS patients with different pTDP-43 pathology stage classifications (stage 1, 2/3 and 4) for each brain region adjusting for identified confounders (Table 2). To identify genes defining each pTDP-43 pathology stage, direct comparisons were made between each stage subgroup and the average of the mean gene expression of the other two stage subgroups [41].

### Pathway analysis of differentially expressed genes

Gene set enrichment analysis (GSEA) was performed using the clusterProfiler package [42]. Differentially expressed genes ranked by *t* statistic (log_2_ fold change divided by standard deviation) and the MSigDB human hallmark gene sets (v2023.1) were used as input. Upstream regulator analysis was performed using QIAGEN IPA (QIAGEN Inc., https://digitalinsights.qiagen.com/IPA) [43]. A cut-off of FDR < 0.05 and|log_2_ fold change| >0.263 (i.e. >20% change in gene expression) was applied to differential gene expression results. Results were filtered for molecules with an|activation z-score| ≥2 and p-value of overlap < 0.05 in at least one brain region and further again for the “transcription regulators” molecule type. Gene ontology (GO) annotation and enrichment analysis for biological process, molecular functions and cellular components was performed using Metascape 3.5 (http://metascape.org) [44]. Default enrichment analysis parameters (minimum overlap = 3, p-value cut-off = 0.01, minimum enrichment = 1.5) were applied.

### Cell-type Deconvolution

RSEM estimated counts were filtered for genes with counts per million (CPM) > 1 in at least 90% of samples. Cortex-derived single cell RNA-seq (scRNA-seq) from Darmanis et al. [45]. and single-nucleus RNA-seq (snRNA-seq) from the Allen Brain Institute (https://portal.brain-map.org/atlases-and-data/rnaseq/human-mtg-smart-seq) were used as reference datasets. Cell-type deconvolution was performed using dtangle v2.0.9 [46] with input counts in log CPM format. Cell-type markers were selected as the top 10% of marker genes identified using the find_markers function with method = “diff”. dtangle cell proportion estimates using the two different reference datasets were correlated to confirm concordance. A second round of deconvolution was performed for non-cortex brain regions using hippocampus [47] and cerebellum snRNA-seq [48] as reference datasets. For the adult hippocampus snRNA-seq, one individual with reactive astrocytosis was removed (Case67) and cell-type subclusters were combined prior to deconvolution. For the adult cerebellum snRNA-seq, control individuals with > 10,000 reads were specifically selected. For both data sets, up to 500 cells were randomly sampled from each cell type for deconvolution. To test for significant ALS-control differences in the estimated cell-type proportions, while accounting for the known significant difference in age, a linear model (proportion ~ disease_status + age) was fitted for each brain region and cell-type combination, and a Bonferroni correction was applied to disease_status p-values. To test for significant differences in cell-type proportions between ALS pTDP-43 pathology stage groups, a Kruskal-Wallis rank sum test was applied.

### Differential transcript usage analysis

To identify transcripts with differential usage in ALS patients relative to controls, IsoformSwitchAnalyzeR v2.0.1 [49, 50] was applied to the RSEM transcript quantification using the DEXSeq implementation of the isoform switch test. Identified confounders were corrected for by inclusion in the design matrix (Table 2). Default parameters were used with significant isoform switches defined as FDR < 0.05 and ≥ 10% change in isoform usage. Genes identified to involve isoform switches were annotated with CPC2 [51], Pfam [52], SignalP [53], NetSurfP-2 [54], analyzeDeepTMHMM [55] and analyzeDeepLoc2 [56]. Isoform switches were predicted to have a functional consequence if annotated protein domains (including intrinsically disordered regions and signal peptides), open reading frames, RNA type (coding, non-coding, nonsense-mediated decay sensitive) or intron retention events were altered between a pair of transcripts with opposing changes in expression (≥10% ALS-control difference).

### Alternative splicing analysis

To identify differential splicing between ALS patients and controls, MAJIQ v2.5.1 [57] was applied to STAR-aligned BAM files. Splice graphs and local splice variations (LSVs) were defined using the MAJIQ builder with GENCODE 43 human reference transcript annotation (gencode.v43.primary_assembly.annotation) and min-experiments = 0.3. MOCCASIN [58] was used to correct MAJIQ output files for identified confounders (Table 2) before performing differential splicing analysis using the MAJIQ heterogen function and min-experiments = 0.3 [59]. Junctions with ΔΨ >10% and Wilcoxon p-value < 0.05 were considered differentially spliced between ALS patients and controls. VOILA was used to obtain MAJIQ differential splicing results (tsv function) and to categorise significant, non-constitutive splicing events (modulize function).

To obtain a high confidence list of genes undergoing differential splicing between ALS patients and controls, LeafCutter v0.2.9 [60] was run in parallel. Importantly, compared to MAJIQ, while LeafCutter is capable of detecting both canonical and *de novo* splicing events it does not detect alternative first and last exons, or intron retention events. We justified that the smaller list of differential splicing events overlapping between MAJIQ and LeafCutter would be more informative, specifically for downstream enrichment analysis. For LeafCutter, junction files were extracted from STAR-aligned BAM files using regtools [61] before performing intron clustering using leafcutter_cluster_regtools.py with default settings. Differential splicing analysis was then performed using leafcutter_ds.R, with the exon file conversion of the GENCODE 43 human reference transcript annotation and including identified confounders (Table 2) as additional columns in the groups_file. Intron clusters with FDR < 0.05 were considered differentially spliced between ALS patients and controls. LeafViz (https://github.com/jackhump/leafviz) was used to categorise splicing events as annotated or cryptic and results were obtained using the export_tables function.

### Detection of reported TDP-43-associated cryptic splicing events

The genomic coordinates of cryptic splicing events associated with TDP-43 nuclear depletion were obtained from three publications examining RNA-seq data from human neuronal models: (1) Neuronal nuclei with and without TDP-43 from FTD-ALS patient post-mortem cortex tissue [27, 62]; (2) Human induced pluripotent stem cell (iPSC)-derived cortical-like i3Neurons with and without reduced TDP-43 expression [26]; (3) iPSC-derived glutamatergic neurons with and without reduced TDP-43 expression [63]. For a cryptic splicing event to be considered detected in our data set, the following criteria had to be met for either MAJIQ or LeafCutter. For MAJIQ, the junction must have ΔΨ > 1% (where ΔΨ is calculated as ALS median Ψ– Control median Ψ), Wilcoxon p-value < 0.05 and de_novo_junction = 1. For LeafCutter, the intron cluster must have FDR < 0.05, while the junction must have ΔΨ > 1% and be classified as unannotated. For cryptic splicing set 1 [27], where the LSV/intron cluster coordinates (i.e. canonical donor and acceptor splice sites) was provided rather than exact cryptic splice site coordinates, at least one junction within the LSV/intron cluster had to satisfy this criterion. We also performed a targeted search for the *STMN2* and *UNC13A* cryptic exons using a previously described bioinformatics pipeline with a detection threshold of 1 count [64].

### Proteomics analysis of post-mortem cerebellum

Post-mortem cerebellum tissue from an extended cohort of 26 sporadic ALS patients (6 stage 1, 10 stage 2–3, 10 stage 4) and 14 controls were used for sequential window acquisition of all theoretical mass spectra (SWATH-MS) analysis. Details of protein sample and local ion library preparation [65], liquid chromatography mass spectrometry (LC-MS) acquisition, and data processing [66] and differential protein expression analysis, can be found in the Supplementary Methods.

## Results

### ALS post-mortem brain RNA-seq cohort

We generated post-mortem brain RNA-seq data for 22 sporadic ALS patients and 11 neurologically normal controls (Table 1). For each individual, five post-mortem brain regions were examined (motor cortex, prefrontal cortex, hippocampus, occipital cortex, and cerebellum; *n* = 165 samples) (Fig. 1a). Significant differences in age at death (*p* = 0.0057) and cerebellar tissue pH (*p* = 0.0049) but not sex, post-mortem delay, or RIN were observed between ALS patients and controls (Figure S3). At the brain region level, no significant ALS-control differences were observed for technical metrics (adjusted p-value < 0.05; Table S4). All ALS patients were further sub-classified by their post-mortem pTDP-43 pathological stage (stage 1 *n* = 6, stage 2–3 *n* = 9, stage 4 *n* = 7). No significant differences in age at death, age at disease onset, site of onset or disease duration were observed between pTDP-43 pathological stage groups (Table S5). While no significant differences in sex were observed between groups, stage 1 ALS included more males (5/6) and stage 4 ALS included more females (5/7) (Figure S4). To confirm that the distribution of brain region gene expression was as expected, principal component analysis (PCA) was performed on the 165 samples and 621 brain RNA-seq samples from the Genotype Tissue Expression Consortium. In both data sets, the first principal component (60–75% variance explained) separated cerebellum samples as a distinct cluster while the second principal component (8–11% variance explained) separated hippocampus and cortex samples with minor overlap (Figure S5).

### Gene expression changes were widespread in ALS post-mortem brain

Differential gene expression analysis that compared ALS patients with controls identified both overlapping and unique gene expression changes in each of the five examined brain regions (Fig. 1b; Table S6). Cerebellum demonstrated the largest number of differentially expressed genes (FDR < 0.05; 3,337 genes), followed by motor cortex (2,236 genes), hippocampus (1,936 genes), prefrontal cortex (1,535 genes) and occipital cortex (1,020 genes). Comparing the expression of 31 ALS-implicated genes [67], *ANG*, *ELP3*, *FUS*, *OPTN*, *PFN1*, *SPG11*, *VAPB* and *VCP* were observed to be differentially expressed between ALS patients and controls across four brain regions (Fig. 1b). *VAPB*, an adaptor protein of the endoplasmic reticulum membrane, was significantly downregulated in the motor cortex, prefrontal cortex, and cerebellum. The largest number of unique differentially expressed genes were observed in the cerebellum (2,007 genes) while the largest overlap in differentially expressed genes occurred between the motor cortex and cerebellum (248 genes; Figure S6). 149 genes were identified to be differentially expressed across all five brain regions, each of which demonstrated concordant direction of change across brain regions. Brain-derived neurotrophic factor (*BDNF*), a neurotrophin with roles in neuronal growth and survival, showed the greatest downregulation across all brain regions in ALS (43–68% reduction of expression in ALS patients). *MT1A*, *ADAMTS9* and *CD163* were among the top upregulated genes across brain regions ranging from 4 to 19-fold increases in ALS patients relative to controls. Correlation of the ALS:control log_2_ fold changes of all tested genes between each brain region identified positive correlations, suggesting shared gene expression changes globally across brain regions (Figure S7a). Motor cortex and prefrontal cortex demonstrated the greatest concordance (*R* = 0.586) while all comparisons with the hippocampus had the weakest associations (R range 0.187–0.350). Positive correlations between brain regions were strong (*R* ≥ 0.7) when we considered only genes with significant differential expression in each region pair (Figure S7b). Among the 63 genes with significant opposing expression between two brain regions was *ATXN1*, which was downregulated in ALS prefrontal cortex and upregulated in ALS cerebellum relative to controls.

Gene set enrichment analysis of 50 hallmark biological processes revealed high concordance across brain regions for ALS-enriched and -depleted pathways (Fig. 1c). Among region-wide enriched pathways were epithelial mesenchymal transition, inflammatory response, hypoxia, and cholesterol homeostasis while oxidative phosphorylation was significantly depleted across all brain regions in ALS patients relative to controls. Few pathways demonstrated discordant normalised-enrichment scores. Of note, genes upregulated in response to interferon alpha were significantly enriched in all brain regions except the occipital cortex. In addition, genes upregulated during the unfolded protein response demonstrated significant depletion in the cerebellum and a positive normalised-enrichment score exclusively in the motor cortex. We next used the differential gene expression results to predict the activity state of upstream regulators of transcription. 82 transcription regulators were predicted to have altered activity in ALS patients relative to controls in at least one brain region (Figure S8). STAT3, HIF1A, JUNB, NFKB1 and SMAD4 were predicted to be significantly activated across all five brain regions while FOXA1, GLI1, NEUROG1, SPDEF and TCF4 were predicted to be inhibited. No transcription regulators showed opposing activity states across the brain regions.

### Cerebellum was predicted to have an altered cell-type composition in ALS patients

**Fig. 2:**
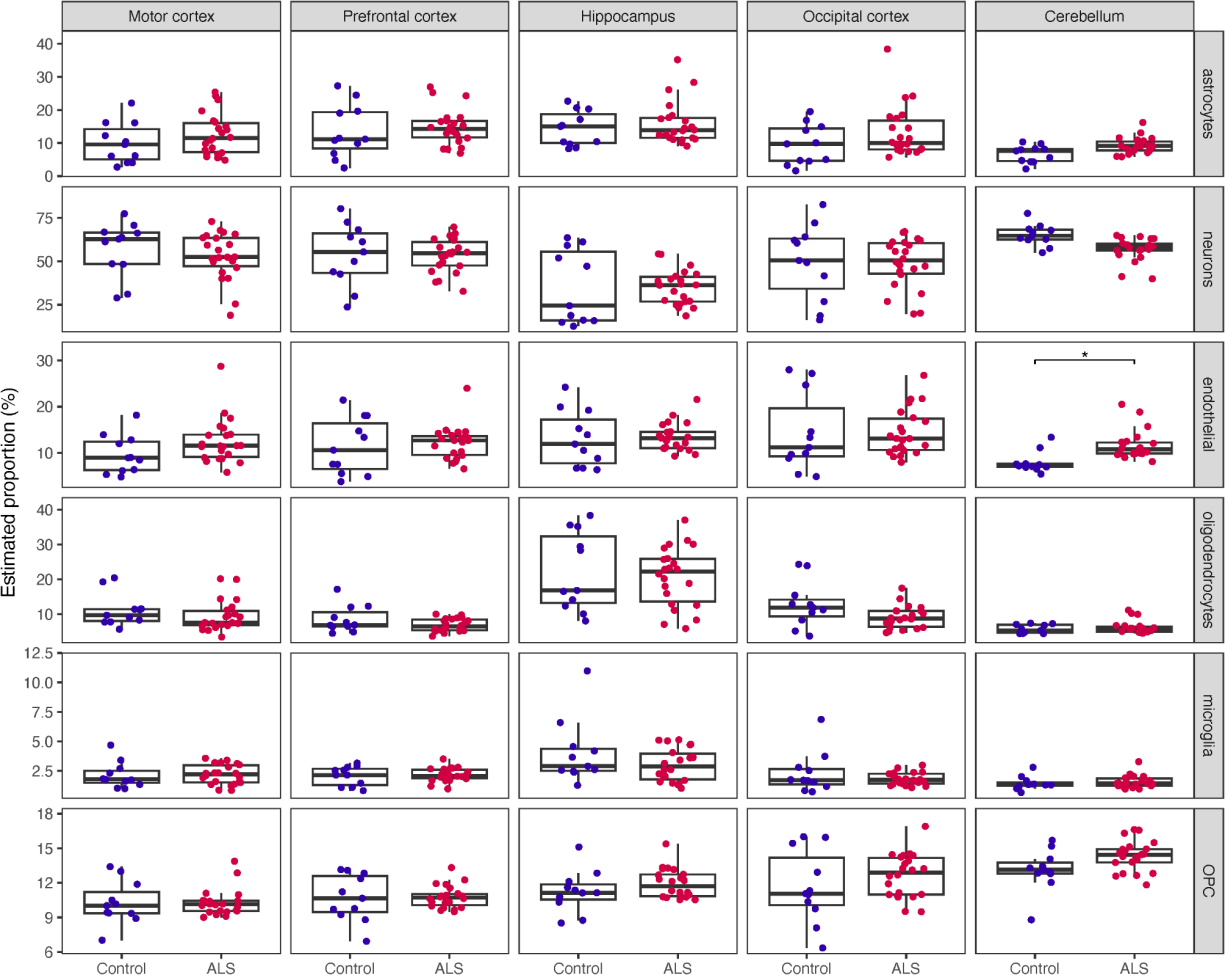
Estimated cell-type proportions across the five brain regions in ALS patients versus controls. Cell-type deconvolution was performed on all 165 brain RNA-seq samples using dtangle. Cortex-derived single cell RNA-seq (Darmanis et al., 2015) was used as the reference data. To test for ALS-control differences in cell type proportions, a linear model (proportion ~ disease_status + age) was fitted for each brain region and cell-type combination, and a Bonferroni correction was applied to disease_status p-values. Cerebellum endothelial cell proportions were significantly different between ALS patients and controls (significance threshold of adjusted p-value < 0.05). OPC, oligodendrocyte progenitor cell

To estimate cellular composition of the ALS and control post-mortem brain tissue samples, cell-type deconvolution was performed using human cortical scRNA-seq as a reference [45]. Across the five examined brain regions, neurons were the most abundant cell type (mean range: 35% [hippocampus]– 60% [cerebellum]), while microglia were the least abundant (mean range: 1.5–3.4%). Neurons (*p* = 0.0023) were predicted to be decreased in ALS patient cerebellum relative to controls, and astrocytes (*p* = 0.0067), oligodendrocyte progenitor cells (*p* = 0.017), and endothelial cells (*p* = 0.0015) were predicted to be significantly increased however, only the latter retained significance following multiple testing correction (Fig. 2; Table S7). A reduction in the motor cortex neuron expression mean (ALS: 53%, Control: 57%) was observed for ALS patients however, this difference was not significant. Addition of the estimated cell-type proportions to our ALS-control differential gene expression models resulted in similar per-gene *t* statistic values to those obtained with the original models (Figure S9). An alternate human cortical snRNA-seq reference (Allen Brain Institute) resulted in similar results whereby cerebellum GABAergic neurons were reduced in ALS cases relative to controls (*p* = 0.022), while astrocytes (*p* = 0.0027) and endothelial cells (*p* = 0.0087) were increased (Figure S10). High concordance (*R* > 0.8) was observed for the estimated cell type compositions between the reference datasets (Figure S11). Comparable estimated cell-type proportions were observed between GTEx brain samples and control samples of the present study (Figure S12).

It has previously been reported that brain region matching between bulk RNA-seq and cell-type reference data is a major determinant of deconvolution accuracy [68]. Therefore, a second round of deconvolution was performed for non-cortex brain regions using hippocampus [47] and cerebellum snRNA-seq [48] as reference datasets. Of eight examined hippocampus cell types, dentate gyrus neurons had a higher mean proportion in ALS patients relative to controls (*p* = 0.012) however, there was a notable split of controls into high and low proportion groups (Figure S13). Of 12 examined cerebellum cell types, Purkinje cells (*p* = 0.0056) and molecular layer interneurons (MLI1, *p* = 0.0066) were predicted to be reduced in ALS patients relative to controls while endothelial cells (*p* = 0.012), astrocytes (*p* = 0.023), Bergman glia (*p* = 0.025) and oligodendrocyte progenitor cells (*p* = 0.026) were predicted to be increased (Figure S14; Table S8). No differences in estimated cell type proportion were significant following multiple testing correction.

### Differential transcript usage events implicated UPR activation and TDP-43 dysfunction

**Fig. 3:**
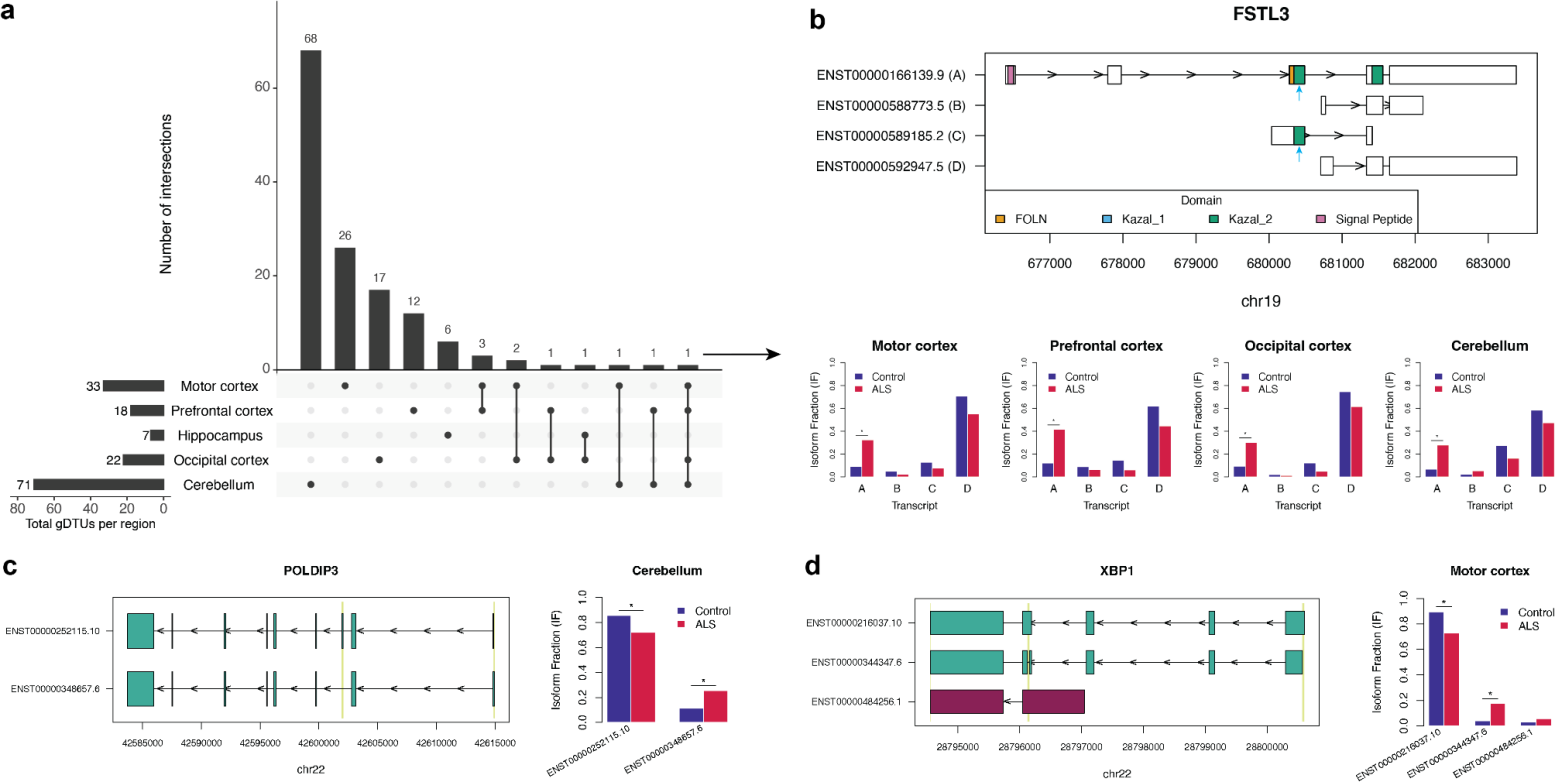
Genes that demonstrated differential transcript usage in ALS patients relative to controls. (a) Overlap of ALS-control differential transcript usage events between the five examined brain regions. In the upset plot the brain regions involved in each intersection are indicated by a filled dot. Significant differential transcript usage events were defined as those with FDR < 0.05 and ≥ 10% change in isoform usage between ALS patients and controls. (b) Differential transcript usage of the canonical *FSTL3* transcript was detected in four brain regions. *Top panel*: Visual depiction of the *FSTL3* transcripts that contributed ≥ 5% of total gene expression in at least one brain region. Predicted Kazal_1 and Kazal_2 domains overlap at blue arrow. *Bottom panel*: Comparison of isoform fractions between ALS patients and controls for each of the four *FSTL3* transcripts. Significant differences (q-value < 0.05) are indicated by an asterisk. Significant differential transcript usage between ALS patients and controls of two isoforms was detected for (c)*POLDIP3* in the cerebellum and (d)*XBP1* in the motor cortex. *Left panels*: Visual depiction of the transcripts that contributed ≥ 5% of total gene expression. Turquoise transcripts are “protein_coding” and maroon transcript is “retained_intron”. The yellow shading behind transcripts highlights the exonic regions that differ between the two significant differentially used transcripts. *Right panels*: Comparison of isoform fractions between ALS patients and controls. Significant differences (q-value < 0.05) are indicated by an asterisk. gDTUs, genes with differential transcript usage

We next examined whether the percentage contribution of a transcript to total gene expression (i.e. transcript usage) was significantly different between ALS patients and controls. Differential transcript usage analysis identified a total of 139 genes with a significant shift in transcript usage in ALS patient brains relative to controls (Table S9). This included differential transcript usage events in 71 genes for the cerebellum, 33 genes for motor cortex, 22 genes for occipital cortex, 18 genes for prefrontal cortex and 7 genes for hippocampus (Fig. 3a). The majority of genes that demonstrated differential transcript usage were not identified as significant differentially expressed genes. Nine genes with differential transcript usage were identified in two brain regions with all differentially used isoforms showing the same direction of change between brain regions. A differential transcript usage event in follistatin-like 3 (*FSTL3*) was detected in all brain regions except the hippocampus (Fig. 3b). This involved a significant ≥ 20% increase in the usage of the canonical transcript encoding a 263 amino acid secreted glycoprotein and a corresponding reduction of a transcript encoding a truncated protein that lacks the follistatin functional domains and is predicted to be located in the cytoplasm.

Of the 139 genes with differential transcript usage, 78 had a pair of transcripts with opposing changes in expression (≥10% ALS: control difference) with 59 of these isoform switches predicted to have a functional consequence (Table S9). For 12 genes, both transcripts involved in the isoform switch were significantly differentially used (Figure S15). Among these was an isoform switch in *POLDIP3* with decreased usage of the canonical transcript (ENST00000252115, q-value = 0.0159) in favour of a transcript excluding exon 3 (ENST00000348657, q-value = 0.0288) in ALS patient cerebellum (Fig. 3c). The exclusion of exon 3 in *POLDIP3* is an established marker of TDP-43 loss of function [69–71]. Notably, a simplified isoform fraction that considered only the expression of the two switched *POLDIP3* transcripts (the canonical and exon 3-excluding transcripts) demonstrated that all five brain regions had increased usage of the exon 3-excluding *POLDIP3* transcript in ALS patients relative to controls (FigureS16a). *XBP1* also demonstrated a dual significant isoform switch with increased usage of a “spliced” transcript (ENST00000344347, q-value = 0.0416) over the alternate “unspliced” transcript (ENST00000216037, q-value = 0.0260) in ALS patient motor cortex (Fig. 3d). The spliced *XBP1* transcript encodes the active form of the transcription factor; a key mediator of the unfolded protein response (UPR), which is increased by inositol requiring enzyme-1 (IRE1) in the presence of endoplasmic reticulum stress [72]. A simplified isoform fraction that considered only the expression of the “unspliced” and “spliced” *XBP1* transcripts demonstrated that the increased usage of the “spliced” *XBP1* transcript in ALS patients was only observed in the motor and prefrontal cortices (Figure S16b).

### Extensive variation in RNA splicing was identified across the ALS brain including cassette exons and intron retention

**Fig. 4:**
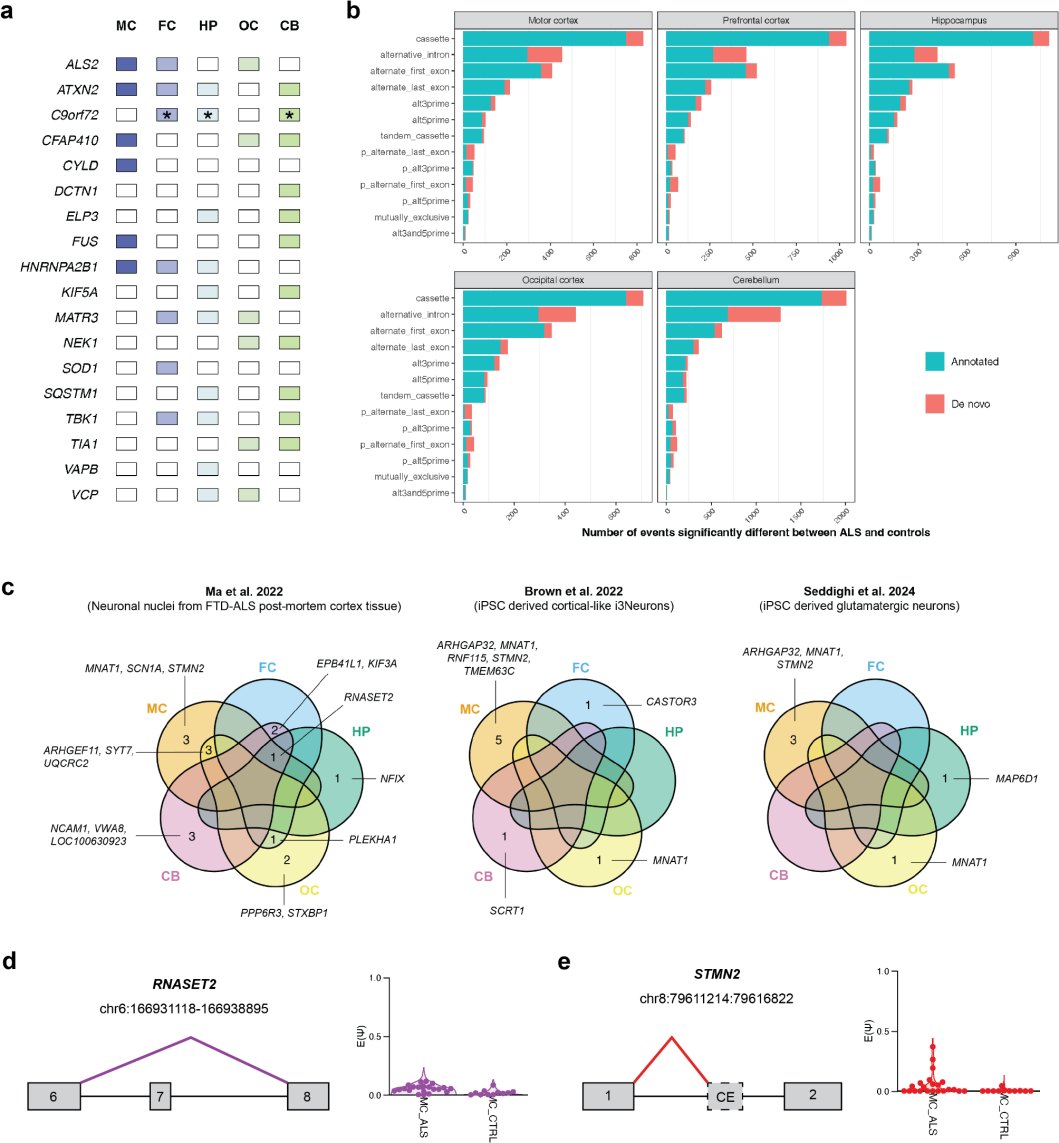
Detection of annotated and cryptic alternative splicing events across ALS patient brain. **(a)** MAJIQ was used to detect transcriptome-wide alternative splicing alterations in ALS patients relative to controls (significance threshold of (ΔΨ > 10%, Wilcoxon p-value < 0.05). Here, ALS-implicated genes that had significant alternative splicing events are highlighted per brain region. Asterix indicates that *de novo* junctions were involved in alternative splicing. ALS-implicated genes with no splicing were *ANG*,* CCNF*,* CHCHD10*,* CHMP2B*,* HNRNPA1*,* NEFH*,* OPTN*,* PFN1*,* SETX*,* SPG11*,* TARDBP*,* TUBA4A* and *UBQLN2*. **(b)** VOILA modulizer categorisation of significant alternative splicing events. See Vaquero-Garcia et al. [59]. for definition of event types. “p_” indicates putative. **(c)** Venn diagram showing the detection of literature-reported cryptic splicing events in each brain region. A cryptic splicing event was considered detected if identified by MAJIQ (ΔΨ > 1%, Wilcoxon p-value < 0.05, de_novo_junction = 1) or LeafCutter (ΔΨ > 1%, FDR < 0.05, classified as unannotated). Visual representation of the **(d)***RNASET2* and **(e)***STMN2* cryptic splicing events. The adjacent violin plot indicates the inclusion level of the of the splice junction [E(Ψ)] in ALS motor cortex (MC_ALS) versus control motor cortex (MC_CTRL), relative to other splice junctions in the same local splicing variation (LSV). MC, motor cortex; FC, prefrontal cortex; HP, hippocampus; OC, occipital cortex; CB, cerebellum

Alternative splicing analysis using MAJIQ, identified between 1,508 (occipital cortex) and 3,586 (cerebellum) local splice variations (LSVs) that were significantly different between ALS patients and controls (ΔΨ >10%, Wilcoxon p-value < 0.05). These LSVs occurred in 2,572 unique genes for the cerebellum, 2,068 for the hippocampus, 1,862 for the prefrontal cortex, 1,522 for the motor cortex and 1,262 for the occipital cortex (Figure S17). A large proportion of the genes identified to have an alternative splicing event were unique to each brain region (range: 26–41%) with only 64 genes in common between all five brain regions. Direct interrogation of 31 ALS-implicated genes [67], identified a significant splicing event between ALS patients and controls for 18 (58%) genes in at least one brain region (Fig. 4a). *ATXN2* demonstrated alternative splicing in the greatest number of brain regions (all but the occipital cortex) while *C9orf72* was the only gene where *de novo* junctions were involved in alternative splicing. To obtain an overview of the types of splicing events that differed between ALS patients and controls, the VOILA modulizer was applied to the MAJIQ heterogen output. Cassette exons were the most frequently detected event type across all brain regions followed by intron retention for the motor cortex, occipital cortex, and cerebellum or alternative first exon for the prefrontal cortex and hippocampus (Fig. 4b). The proportion of events with a *de novo* junction ranged from 14.0% in the hippocampus to 23.6% in the cerebellum. Notably, almost half (46%) of all intron retention events in the cerebellum were classified as *de novo*. To determine whether genes with particular biological roles were the target of alternative splicing in ALS patients, first, a high confidence list of alternatively spliced genes was obtained by taking the overlap of significant genes identified by both MAJIQ and LeafCutter (FDR < 0.05) per brain region (Table S10). Gene Ontology (GO) enrichment analysis of these reduced gene lists revealed many terms that were neuron, cytoskeleton and/or cell junction related across the five brain regions (Figure S18). Also recurring was RNA splicing related terms in the motor cortex and prefrontal cortex, and vesicle transportation in the hippocampus, occipital cortex and cerebellum.

### Cryptic splicing events associated with nuclear depletion of TDP-43 were detected in multiple brain regions

**Fig. 5:**
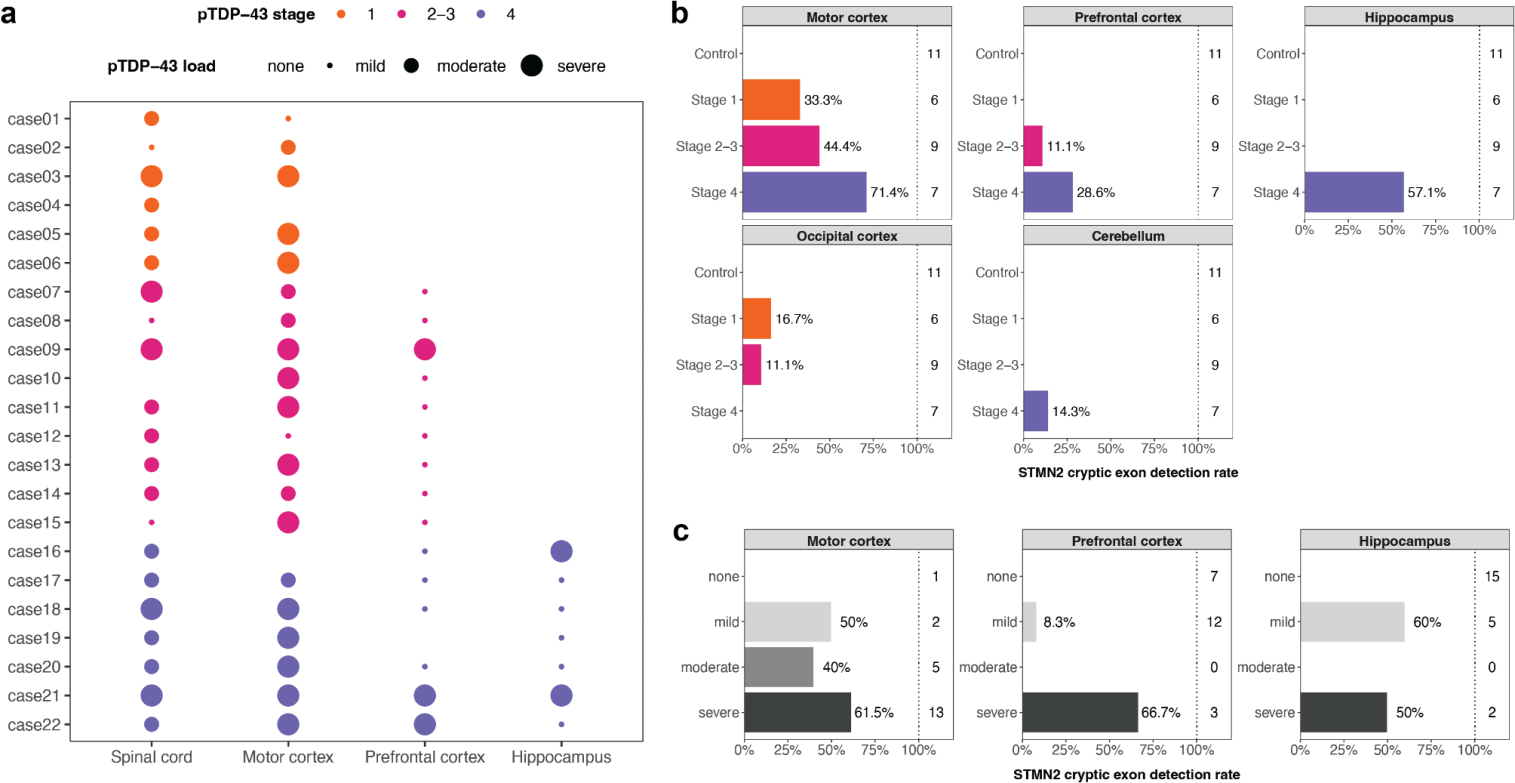
*STMN2* cryptic exon detection in the context of pTDP-43 pathological stage and load. **(a)** pTDP-43 pathology stage classification for the 22 ALS patients examined by RNA-seq and their semi-quantitative pTDP-43 pathology load for four examined CNS regions. The pTDP-43 pathology load was missing for Case16 in the motor cortex. Detection of the *STMN2* cryptic exon by: **(b)** pTDP-43 pathology stage group and **(c)** pTDP-43 pathology load. Only one read spanning the *STMN2* cryptic exon junction (chr8:79611214:79616822) was required to be called as detected. The number of individuals within each group is shown to the right of the dotted line

Next, we examined whether cryptic splicing events previously reported to be associated with TDP-43 nuclear depletion in human post-mortem [27] or iPSC-derived neurons [26, 63], could be detected in our brain RNA-seq. A total of 645 cryptic splicing events across 349 genes were examined (Table S11), with a cryptic splicing event considered to be detected if it was identified by MAJIQ (ΔΨ > 1%, Wilcoxon p-value < 0.05, de_novo_junction = 1) or LeafCutter (ΔΨ > 1%, FDR < 0.05, classified as unannotated). The low ΔΨ threshold was selected because neuron attributable reads are expected to be diluted in bulk RNA-seq. Literature-reported cryptic splicing events in 22 genes were identified in our brain RNA-seq, seven of which were present in more than one brain region (Fig. 4c). Notably, the junction coordinates of 207 (32%) of the reported cryptic splicing events were detected in our brain RNA-seq however, the majority did not have significant differential usage between ALS patients and controls or were not classified as cryptic (Figure S19). A cryptic splicing event in *RNASET2* (ribonuclease T2) that leads to exclusion of exon 7 was present in all brain regions but the occipital cortex (Fig. 4d). The inclusion of a cryptic exon in *STMN2* (stathmin 2), a well-studied cryptic splicing event that arises from TDP-43 loss of function [24, 25], was identified to have significantly increased in the motor cortex (Fig. 4e). The *STMN2* cryptic exon has also been reported to be significantly associated with pTDP-43 levels in frontotemporal lobar degeneration (FTLD) [64]. To examine this in our own cohort, we performed targeted detection of the *STMN2* cryptic exon per individual and aligned this with our pTDP-43 stage classification and pathology load (i.e. semi-quantitative pTDP-43 burden per brain region) (Fig. 5a). The *STMN2* cryptic exon was detected in the motor cortex of half of all ALS patients, with an increasing detection rate observed from pTDP-43 pathology stages 1 to 4 (Fig. 5b). For the other two brain regions where pTDP-43 pathology load information was available (prefrontal cortex and hippocampus), the *STMN2* cryptic exon was exclusively detected in individuals with pTDP-43 pathology in those regions (*n* = 3 in prefrontal cortex, *n* = 4 in hippocampus). The *STMN2* cryptic exon was also detected in the occipital cortex of one stage 1 and one stage 2–3 ALS case, and in the cerebellum of one stage 4 ALS case but was absent from controls across all five brain regions. When the observed pTDP-43 pathology load was considered, we found that the *STMN2* cryptic exon could be detected in ALS patients with mild to severe pTDP-43 pathology (Fig. 5c). We were unable to detect the previously reported *UNC13A* cryptic exon inclusion events in our samples using the same targeted search approach. However, *UNC13A* expression was significantly downregulated in ALS motor cortex in line with previous reports that, unlike the *STMN2* cryptic exon, the *UNC13A* cryptic exon induces nonsense-mediated decay [26, 27].

### Stage 4 ALS patients had a distinct gene expression signature in the cerebellum

**Fig. 6:**
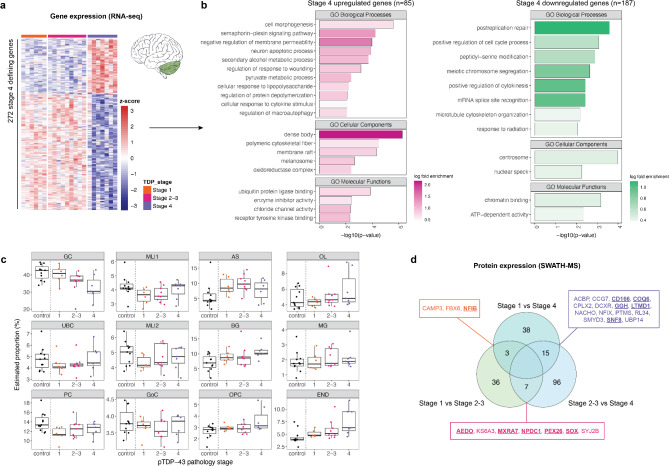
Stage 4 ALS cerebellum demonstrated a distinct gene expression profile. **(a)** Heatmap of the 272 genes identified to be significantly different in stage 4 ALS patient cerebellum relative to stages 1–3. Gene counts are z-score normalised. **(b)** GO terms enriched among the genes upregulated and downregulated in stage 4 ALS patient cerebellum. The most representative member (lowest p-value) of each GO term cluster is displayed. Log_2_ fold change indicates enrichment of member genes in differentially expressed genes versus all detected genes. **(c)** Cell-type deconvolution result using dtangle and cerebellum-derived single-nucleus RNA-seq (Tejwani et al., 2024). No cell type proportions were significantly different between ALS pTDP-43 pathology stage groups (Kruskal-Wallis rank sum test; significance threshold of p-value < 0.05). Controls were not included in statistical testing and are included here purely as a reference. **(d)** Venn diagram showing the number of proteins detected by SWATH-MS as differentially expressed in the cerebellum between each ALS pTDP-43 pathology stage group. Proteins defining each stage (i.e. the overlap of proteins identified as significantly up- or downregulated from each comparison) are indicated in boxes. Upregulated proteins are underlined while downregulated proteins appear in regular text. GC, granule cells; UBC, unipolar brush cells; PC, Purkinje cells; MLI1, molecular layer interneuron population 1; MLI2, molecular layer interneuron population 2; GoC, Golgi cells; AS, astrocytes; BG, Bergmann glia; OPC, oligodendrocyte progenitor cells; OL, oligodendrocytes; MG, microglia; END, endothelial cells

Considering the role of TDP-43 in RNA regulation, we next aimed to determine whether the post-mortem pTDP-43 pathology staging scheme reflected not only the regional occurrence of pathology but also transcriptome differences between the stages. To examine whether ALS patients with different pTDP-43 pathology stages could be distinguished based on gene expression, direct comparisons were made between each stage subgroup for each brain region. In cerebellum, 272 genes were significantly differentially expressed between stage 4 and stage 1–3 ALS patients (Table S12). For the remaining brain regions, no differentially expressed genes were detected between the stage subgroups other than two genes downregulated in stage 2–3 ALS motor cortex (*CRH*, *PMAIP1*). Heatmap visualisation of the 272 genes in the cerebellum that distinguish the stage 4 subgroup from stages 1–3 subgroup highlighted a distinct gene expression signature for the stage 4 ALS patients (Fig. 6a). Enrichment analysis of stage 4 ALS cerebellum upregulated genes (*n* = 85) and downregulated genes (*n* = 187) identified an overrepresentation of a small number of GO terms (Fig. 6b). GO biological pathways enriched in stage 4 ALS upregulated genes broadly captured metabolic processes, inflammatory response, and cell development, the latter of which represented clusters including neuron-specific terms (Table S13). The top ranked GO molecular function was ubiquitin protein ligase binding while the top cellular component was dense body, a high-level term representing cytoskeletal changes. GO terms enriched in stage 4 ALS downregulated genes were largely related to cell division, DNA damage repair and RNA splicing. We next considered whether estimated cell-type proportions were variable between ALS pTDP-43 pathology stage groups and found no significant differences for any of the five examined brain regions using the human cortical scRNA-seq reference (Figure S20). Deconvolution results using the cerebellum snRNA-seq as reference also did not identify any significant differences in cerebellum cell-type proportions between pTDP-43 pathology stage groups (Fig. 6c). Of note, the deviation of estimated cell-type proportions from control levels was variable between pTDP-43 pathology stage groups, with select cell types such as the granule cells and endothelial cells, demonstrating a progressive decline or increase in proportion means across the stages.

To determine whether gene expression changes observed between pTDP-43 pathology stage subgroups translated to the protein level, SWATH-MS was performed on ALS post-mortem cerebellum. These samples overlapped with the RNA-seq cohort but included one additional stage 2 case and three additional stage 4 cases (Table S14). No significant differences in demographic, clinical or technical metrics were observed between ALS stage subgroups used for SWATH-MS (Figure S21; Table S15). Of the 3,516 proteins detected by SWATH-MS, 195 proteins were found to be significantly differentially expressed in at least one comparison between stage subgroups (Table S16). When taking the overlap of pairwise comparisons, three differentially expressed proteins defined stage 1 ALS patients, seven for stage 2–3 ALS patients and 15 for stage 4 ALS patients (Fig. 6d). None of these stage defining proteins overlapped with stage 4 ALS differentially expressed genes, and gene and protein fold changes for each pairwise comparison were not correlated overall (Figure S22).

## Discussion

In this study, we performed a multi-regional transcriptomic analysis of ALS post-mortem brain to improve our understanding of broader neuropathological changes associated with the disease. Importantly, we examined individual-matched samples to maximise the informativeness of inter-regional comparisons, which may otherwise be obscured by disease heterogeneity. Our results highlighted widespread changes in RNA expression and splicing in the ALS brain, some of which are a known consequence of TDP-43 dysregulation. Cerebellum exhibited the largest alterations across analyses, implicating a strong involvement in disease, with cerebellar gene expression changes also influenced by the regional spread of pTDP-43 pathology elsewhere in the brain.

The findings from this study shine a spotlight on the cerebellum, a region whose involvement in ALS is contentious [73]. Previously reported ALS post-mortem RNA-seq studies characterised the cerebellum transcriptome in the context of *C9orf72* genotype [17, 20]. Each study reported substantially greater cerebellum gene expression and alternative splicing alterations in individuals who carried a pathogenic *C9orf72* repeat expansion. In the present study we confirmed that changes in the cerebellum transcriptome were also present in sporadic ALS patients without disease-associated repeat expansions in *C9orf72* and *ATXN2*. Surprisingly, the scale of changes observed in ALS cerebellum was greater than seen in other brain regions that are considered more disease relevant. Under the assumption that neurons are the most disease-relevant cell type, a possible contributing factor to the strong cerebellum signal is the high neuronal density of the cerebellum, which houses approximately 80% of all neurons in the brain [11, 74]. Moderate shifts in ALS patient cerebellum cell-type proportions, as predicted by cell-type deconvolution, may have also increased the observed gene and transcript expression changes seen in this region. Of note, while we report a significant reduction in cerebellum tissue pH in ALS patients relative to controls, cerebellum is an established proxy tissue for overall brain pH, and as such, is the only region routinely used for pH measurement [75–78]. Progressive reduction in brain pH has previously been associated with aging and neurodegenerative disease [79], and we observed significant enrichment and depletion of hypoxia and oxidative phosphorylation gene expression, respectively, across all five brain regions in ALS. We did not identify any other technical causes to explain the considerable changes observed in the ALS cerebellum transcriptome however, the increased prevalence of differential transcript usage and alternative splicing events could also be partly attributed to the higher transcript diversity of the cerebellum relative to other brain regions [80]. Nevertheless, the extensive transcriptomic changes seen here in sporadic ALS cerebellum are intriguing and supports calls to re-evaluate the cerebellar role in ALS pathogenesis and progression [73, 81].

In the present study we observed largely concordant pathway enrichment and predicted transcription factor activity across five brain regions variably affected by pTDP-43 pathology in ALS. A recent multi-region post-mortem RNA-seq study of FTLD with TDP-43 pathology similarly reported common enrichment of biological pathways and cell types in FTLD between the primary affected frontal and temporal cortices and the cerebellum [82]. Highly conserved transcriptomic changes have also been described between the temporal cortex (a region that presents with tau pathology) and cerebellum (a largely pathologically unaffected region) in Alzheimer’s disease and progressive supranuclear palsy [83]. Comparably to these other studies, we observed positive correlations of ALS:control gene expression fold changes between brain regions, indicative of shared transcriptome alterations. The conserved gene expression changes observed in brain regions variably affected by gross neuropathology suggest the presence of an upstream, brain-wide pathology, triggering common pathological or compensatory responses in these neurodegenerative diseases [83, 84]. Furthermore, the apparent absence of overt pTDP-43 inclusions and neurodegeneration in regions like the cerebellum and occipital cortex, suggests that they may be protected or resistant to the pathological sequelae of ALS. Further examination of these lesser-studied brain regions is warranted to better understand the molecular mechanisms underlying selective neuroanatomical vulnerability. Neuroanatomical differences were also evident in our ALS transcriptomes, in particular when considering transcript-level analyses, with a substantial proportion of differential transcript usage and alternative splicing events demonstrating regional specificity. For example, significantly increased usage of the spliced *XBP1* isoform and positive normalised enrichment score for unfolded protein response genes was specific to the motor cortex. Activation of the unfolded protein response via the IRE1/XBP1 pathway has previously been reported in sporadic ALS motor cortex [85], with our findings now suggesting that this activation is brain region-specific. Importantly, it is difficult to delineate many of the observed region-specific transcriptional changes from inter-region variance in cell-type composition. Emerging single cell RNA-seq studies of ALS post-mortem tissue will be critical to clarifying the cell-type origin of observed transcriptomic changes and improving our understanding of regional response to disease [86–88]. Further, we acknowledge that the examined cohort may not capture the complete transcriptome diversity of both ALS and older control populations. Nevertheless, it is evident that the neurodegenerative process has a broad impact across the ALS brain, extending beyond sites of visible pathology and questioning whether any brain region may be considered truly unaffected in ALS.

The regulation of RNA processing is an established function of TDP-43, with transcript mis-splicing demonstrated to be a consequence of its depletion from the nucleus and/or loss of function [69, 89]. We found that aberrant RNA splicing events associated with TDP-43 dysregulation were not restricted to brain regions with hallmark pTDP-43 inclusion pathology. We observed a significant increase in *POLDIP3* exon 3 exclusion in ALS cerebellum relative to controls, with the other four brain regions showing a similar but non-significant trend. Increased *POLDIP3* exon 3 exclusion is an established marker of TDP-43 loss of function which has been validated post-mortem in ALS motor cortex, spinal cord and spinal motor neurons [71], and in ALS/FTD neuronal nuclei with loss of nuclear TDP-43 [62, 69]. Furthermore, TDP-43-associated cryptic splicing events [26, 27, 63] were detected across all five examined brain regions. Previous examination of the *STMN2* and *UNC13A* cryptic exons in bulk CNS RNA-seq found that they were largely detected in the motor cortex and spinal cord of ALS cases, and the frontal and temporal cortices of FTLD cases, the primary sites of pTDP-43 pathology [26, 27, 64]. Other examined regions in these studies, including the hippocampus, occipital cortex and cerebellum, had low to no cryptic exon detected. Standard-depth bulk RNA-seq of post-mortem tissue is not the typical method for the detection of cryptic splicing events, which are expected to be present in the small proportion of pathology-affected cells remaining at end-stage disease. Yet our study has demonstrated that bulk RNA-seq can indeed detect cryptic splicing events. Pickles et al. [84]. utilised targeted NanoString analysis to identify significantly elevated levels of *STMN2* cryptic exon-containing RNA in post-mortem cerebellum tissue from FTLD patients with TDP-43 pathology. They further demonstrated that *STMN2* cryptic exon-containing transcripts were inversely associated with detergent-soluble TDP-43 protein levels in the cerebellum and critically, that the reduction in detergent-soluble TDP-43 observed in FTLD cases was not accompanied by a concomitant increase in detergent-insoluble (i.e. aggregated) TDP-43 [84]. This suggested that not only was TDP-43 dysfunctional in the cerebellum but also that the apparent loss of TDP-43 function is uncoupled from overt pTDP-43 inclusion pathology. Supporting the latter notion, cryptic exons have been detected in Alzheimer’s disease hippocampus tissue that exhibited TDP-43 nuclear clearance without inclusion pathology [90]. Furthermore, a recent study that employed a TDP-43 RNA aptamer described a nuclear puncta TDP-43 pathology in ALS frontal cortex tissue that was associated with coincident *STMN2* cryptic splicing [91]. The authors proposed that nuclear TDP-43 pathology represented an early pathological event preceding both cytoplasmic TDP-43 pathology and region-specific clinical manifestation. Our own observations of TDP-43-associated splicing events in five ALS post-mortem brain regions suggests that more subtle markers of early pathological events, including TDP-43 dysfunction, are likely present in broader neuroanatomical regions. The large number of alternative splicing events observed in ALS post-mortem tissue also suggests that other regulators of RNA splicing, in addition to TDP-43, are dysfunctional. Cryptic splicing discovery studies have focused on more homogenous sample types such as neuronal cell lines or iPSC-derived neurons with TDP-43 depletion [26, 63, 89]. It is unclear whether many of the literature-reported cryptic splicing events translate from model system to patient and whether they are conserved across different genetic backgrounds. To this point, a study examining *C9orf72*-positive ALS/FTD frontal and occipital cortex using single-nuclei RNA-seq was only able to detect increased expression of two (*STMN2*, *KALRN*) of the 66 previously reported post-mortem TDP-43-associated cryptic splicing events [27] also examined in the present study [92]. While technical limitations have played a role in this variable detection, clarifying the cryptic splicing landscape in the ALS brain will be critical to understand the upstream triggers and downstream consequences of alternative slicing events, and will help identify specific events that represent promising therapeutic targets [93].

The ALS post-mortem pTDP-43 pathology staging scheme supports the hypothesis of pathological spread via axonal pathways [13, 14] however, it is unclear whether the variable regional presence of pTDP-43 pathology is associated with discrete patient subgroups. We observed differential gene expression between stage 1–3 and stage 4 ALS patients, specifically in the cerebellum. Though we did not identify any statistically significant clinical differences between pTDP-43 pathological stage subgroups in our study, another study that supported the staging scheme reported a significantly later age of onset for stage 4 ALS patients [14]. Fatima et al. also observed that stage 4 ALS cases had a shorter disease duration than stage 3 cases but similar disease duration to stage 2 cases, suggesting that stage 4 cases may not be on the same disease continuum. ALS patients who carried a pathogenic *C9orf72* repeat expansion have been reported to have a greater regional burden of pTDP-43 pathology [13, 94]. Although they were not included in this study, all eleven *C9orf72*-positive ALS cases in the Sydney Brain Bank were classified as stage 4. There have been previous reports of a sporadic ALS patient subgroup with molecular [19] and neuroimaging [95] phenotypes congruent to *C9orf72*-positive ALS patients. Whether stage 4 pTDP-43 pathological classification underlies shared features between a sporadic ALS patient subgroup and *C9orf72*-positive ALS patients requires further investigation. The cohort used in the present study is limited by size when sub-categorised by pTDP-43 pathological stage and *C9orf72*-positive ALS cases were not co-examined. Re-examination of published ALS/FTD post-mortem cerebellum RNA-seq cohorts [17, 20], with consideration of pTDP-43 pathological staging (namely the occurrence of hippocampal pathology), could address this question.

## Conclusion

Our analysis identified widespread RNA alterations in sporadic ALS post-mortem brain. We observed aberrant TDP-43-associated splicing events across five brain regions that variably presented with pTDP-43 inclusions, suggesting that TDP-43 dysfunction extends beyond sites of overt hallmark pTDP-43 neuropathology. Furthermore, conserved gene expression changes between spatially distinct brain regions and unexpected pathology-associated changes in the cerebellum transcriptome, support the notion that ALS is a multisystem neurodegenerative disease in which some brain regions are more vulnerable to the ALS pathological sequelae.

## Electronic supplementary material

Below is the link to the electronic supplementary material.

Supplementary Material 1: Supplementary tables: Contains tables S1 to S16.

Supplementary Material 2: Figures S1 to S22 and supplementary methods.

## Data Availability

Raw FASTQ files have been deposited on The National Institute on Aging Genetics of Alzheimer’s Disease Data Storage Site (NIAGADS) under accession number NG00178 (10.60859/kp1c-rg36). Code written in R is available in R Markdown workbooks in a GitLab repository: https://gitlab.com/mq-mnd/grp_williams/als_brain_transcriptome_pub. The authors would also like to acknowledge publicly available code [21, 64, 82] that aided figure generation.

## Abbreviations

ALS: Amyotrophic lateral sclerosis
BIC: Bayesian information criterion
CNS: Central nervous system
CPM: Counts per million
FDR: False discovery rate
FTD: Frontotemporal dementia
FTLD: Frontotemporal lobar degeneration
GO: Gene ontology
GSEA: Gene set enrichment analysis
GTEx: Genotype tissue expression consortium
iPSC: Induced pluripotent stem cell
IRE1: Inositol requiring enzyme-1
LC-MS: Liquid chromatography mass spectrometry
LSV: Local splice variation
PCA: Principal component analysis
pTDP-43: Phosphorylated TAR DNA-binding protein
RIN: RNA integrity number
RNA-seq: RNA sequencing
SCA2: Spinocerebellar ataxia 2
scRNA-seq: Single cell RNA sequencing
snRNA-seq: Single nuclei RNA sequencing
SWATH-MS: Sequential window acquisition of all theoretical mass spectra
UPR: Unfolded protein response
