# Supplementary material for: Multi-region brain transcriptomic analysis of amyotrophic lateral sclerosis reveals widespread RNA alterations and substantial cerebellum involvement
Source: Mol Neurodegener. 2025 Apr 25;20:40. doi: 10.1186/s13024-025-00820-5 (PMC12023386; doi:10.1186/s13024-025-00820-5)
Supplement: Supplementary file 2 — Supplementary Material 2: Figures S1 to S22 and supplementary methods. [file 13024_2025_820_MOESM2_ESM.pdf]

Supplementary Materials for

Multi-region brain transcriptomic analysis of amyotrophic lateral sclerosis reveals widespread RNA alterations and substantial cerebellum involvement

Natalie Grima, Andrew N. Smith, Claire E. Shephard, Lyndal Henden, Thiri Zaw, Luke Carroll, Dominic B.Rowe, Matthew C. Kiernan, Ian P. Blair and Kelly L. Williams*

*Corresponding Author. Email: kelly.williams@mq.edu.au

This PDF file includes:

Figs. S1 to S22

Supplementary Methods

Supplementary Figures

S1 Overview of the RNA-seq data processing and analysis pipeline used in this study 4
S2 variancePartition result for independent variables 5
S3 Comparison of key samples features between ALS patients (n=22) and controls (n=11) 8
S4 Comparison of clinical features between ALS patient pTDP-43 pathology stage groups 9
S5 Comparison of the current study's brain gene expression distribution to the Genotype Tissue Expression (GTEx) Consortium 10
S6 Overlap of ALS-control differentially expressed genes between the five examined brain regions 11
S7 Correlation of ALS:control $\log_2$ fold changes between each brain region 12
S8 Transcription regulators predicted to be activated or inhibited in ALS patients relative to controls 13
S9 Comparison of per-gene t statistic values when estimated cell-type proportions are added to ALS-control differential gene expression models 14
S10 Estimated proportions of seven major cell types in ALS patients versus controls across all five brain regions 15
S11 Correlation of dtangle estimated proportions of six major cell types for Darmanis versus Allen Brain Institute reference data 16
S12 Estimated cell-type proportions for Genotype Tissue Expression (GTEx) Consortium brain samples versus controls 17
S13 Estimated proportions of eight hippocampus cell types in ALS patients versus controls 18
S14 Estimated proportions of 12 cerebellum cell types in ALS patients versus controls 19
S15 Visual of 12 genes identified to have differential transcript usage of two isoforms between ALS patients and controls 20
S16 Simplified isoform fraction of *POLDIP3* and *XBP1* differentially used transcripts 24
S17 Overlap of ALS-control alternatively spliced genes between the five examined brain regions as determined by MAJIQ 25
S18 Gene ontology (GO) enrichment analysis result for genes identified as differentially spliced between ALS patients and controls 26
S19 Detection of literature-reported cryptic splicing events in bulk brain RNA-seq data 29
S20 Estimated proportions of six major cell types in ALS patient pTDP-43 pathology patient subgroups across all five brain regions 30
S21 Comparison of samples features between ALS patient pTDP-43 pathology stage groups (SWATH-MS cohort) 31
S22 Correlation of cerebellum gene and protein $\log_2$ fold changes for each pTDP-43 pathology stage pairwise comparison 32

Supplementary Methods

Cohort selection **33**

Cell-type deconvolution **33**

Alternative splicing analysis **33**

LeafCutter Docker container 33

Proteomics analysis of post-mortem cerebellum **33**
Protein sample and local ion library preparation 33
LC-MS acquisition 34
Proteomics data processing and differential protein expression analysis 34

Supplementary Figures

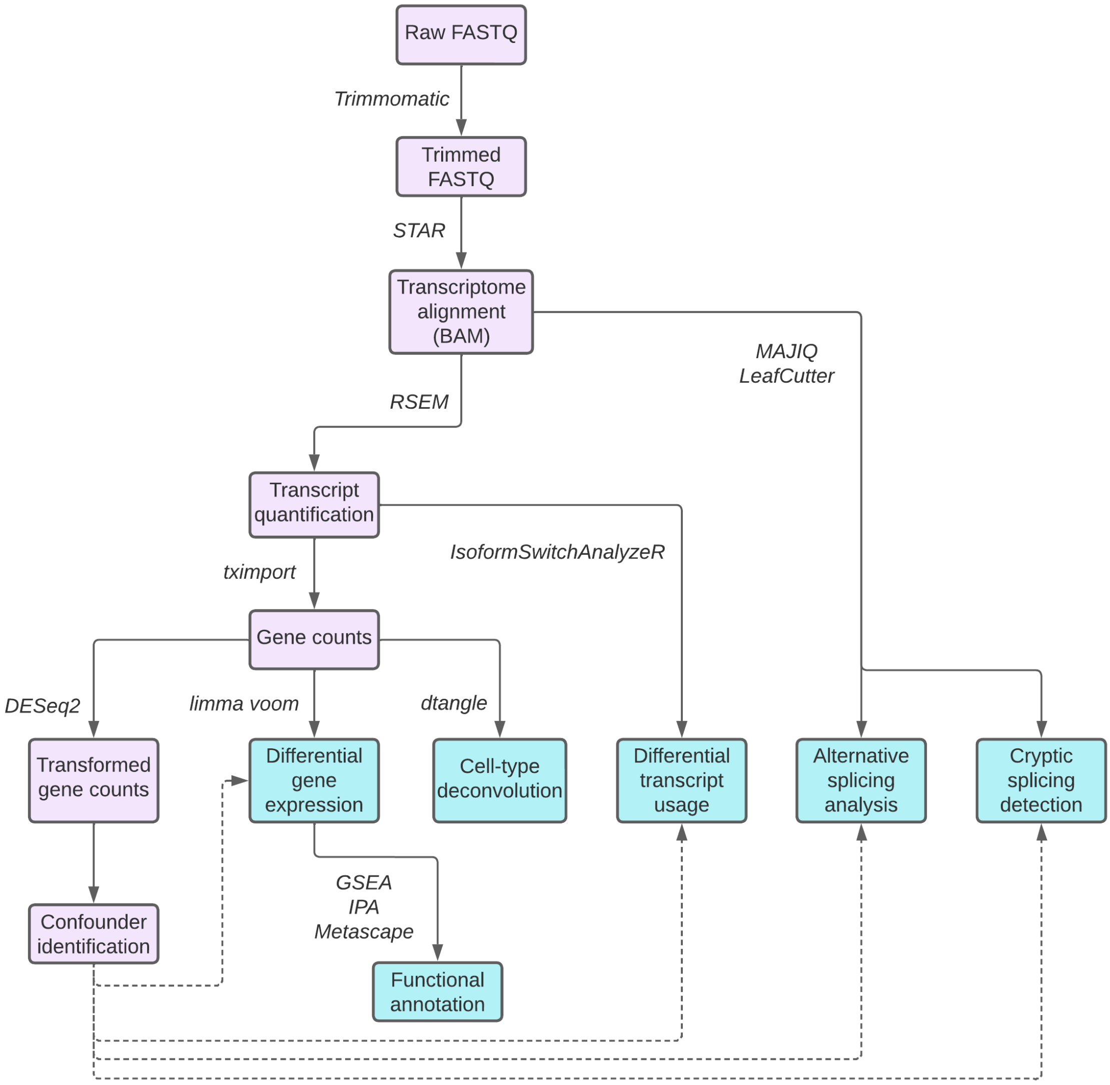

Fig. S1: Overview of the RNA-seq data processing and analysis pipeline used in this study. RNA-seq data processing and analyses are highlighted in purple and blue, respectively. The bioinformatic tool or package used at each step is listed in italics to the left of each arrow. Dashed lines highlight the analyses that accounted for identified confounders.

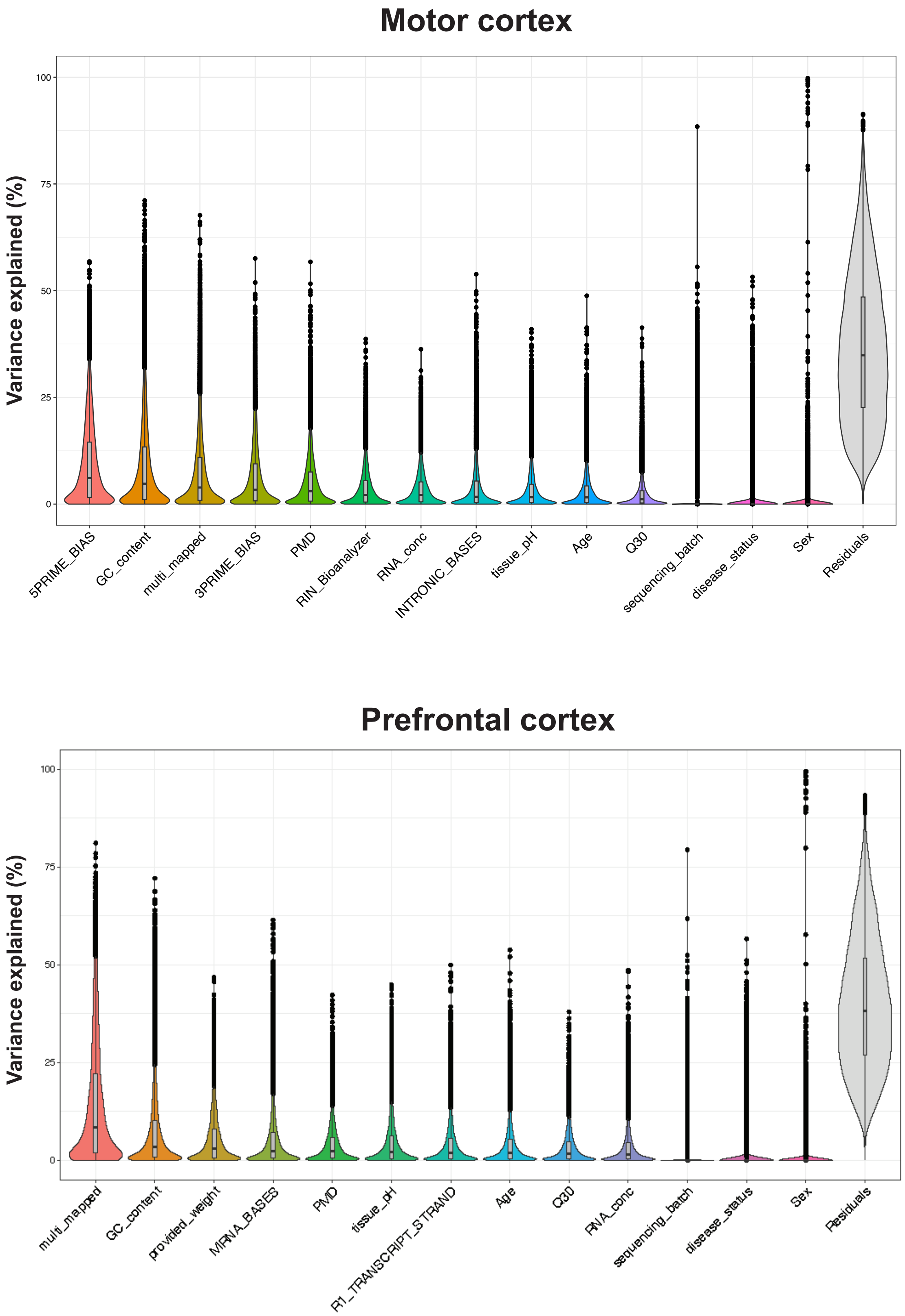

Fig. S2: variancePartition result for independent variables. Following the identification of independent covariates and factors, variancePartition was used to identify the five variables with the greatest contribution to total variance. The complete confounder identification procedure is provided in Table S3.

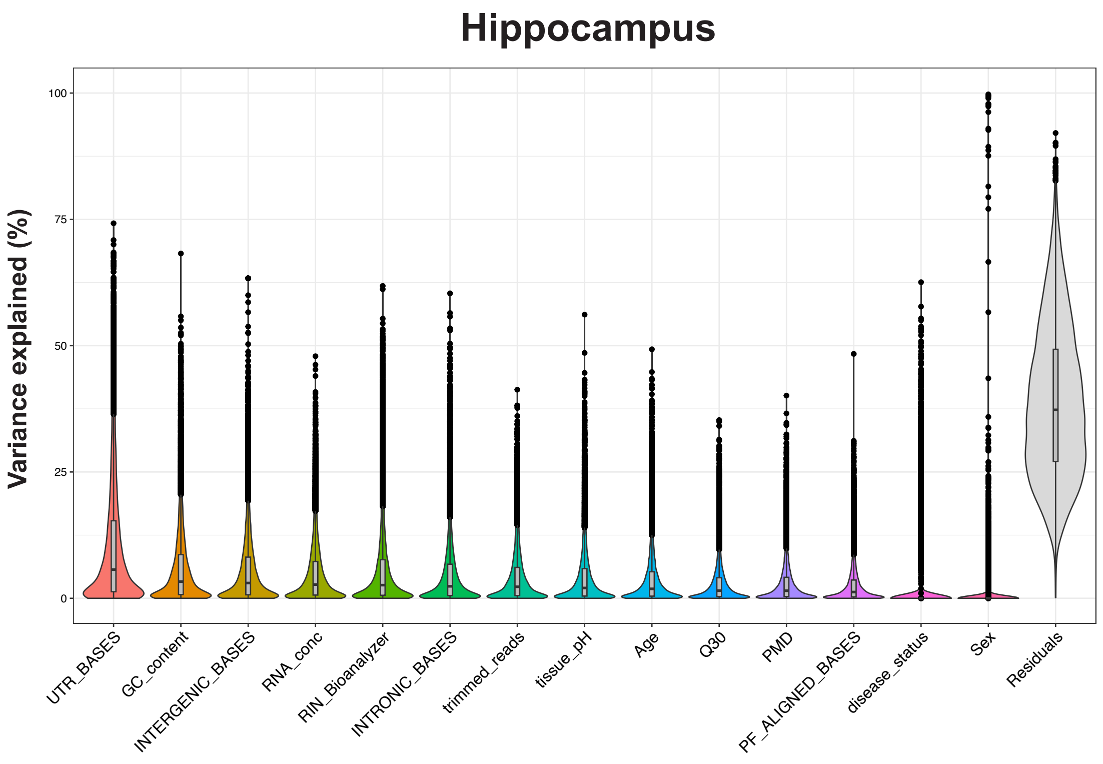

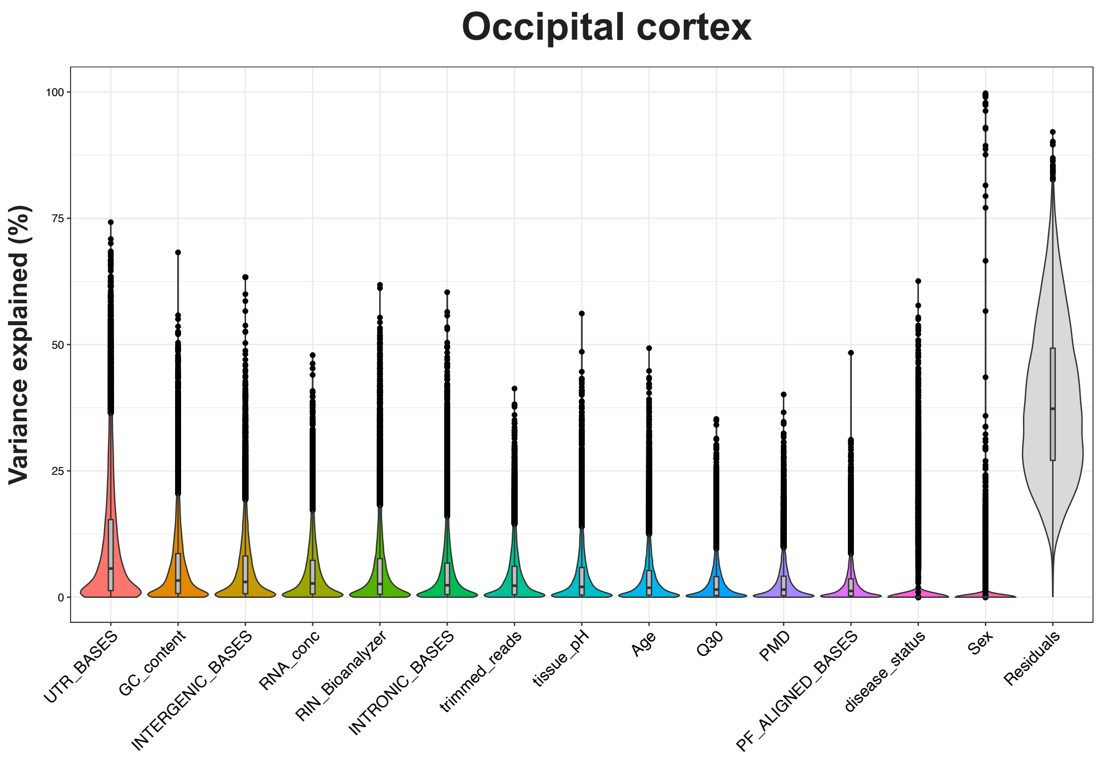

Fig. S2 (continued): variancePartition result for independent variables. Following the identification of independent covariates and factors, variancePartition was used to identify the five variables with the greatest contribution to total variance. The complete confounder identification procedure is provided in Table S3.

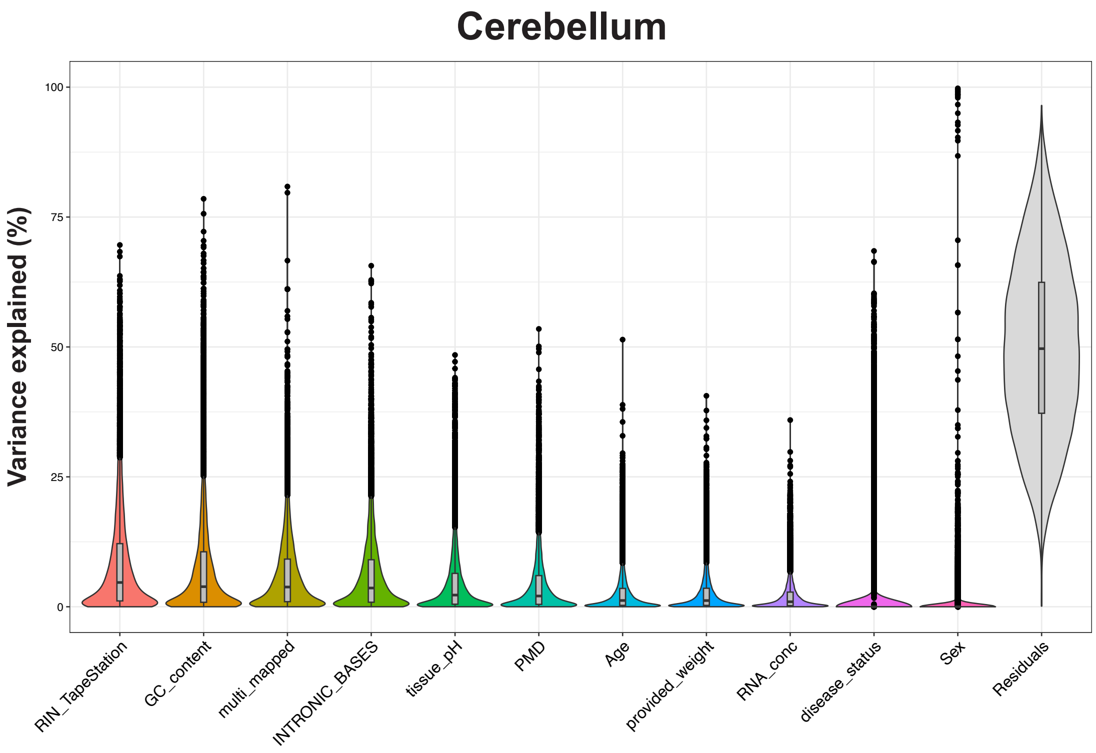

Fig. S2 (continued): variancePartition result for independent variables. Following the identification of independent covariates and factors, variancePartition was used to identify the five variables with the greatest contribution to total variance. The complete confounder identification procedure is provided in Table S3.

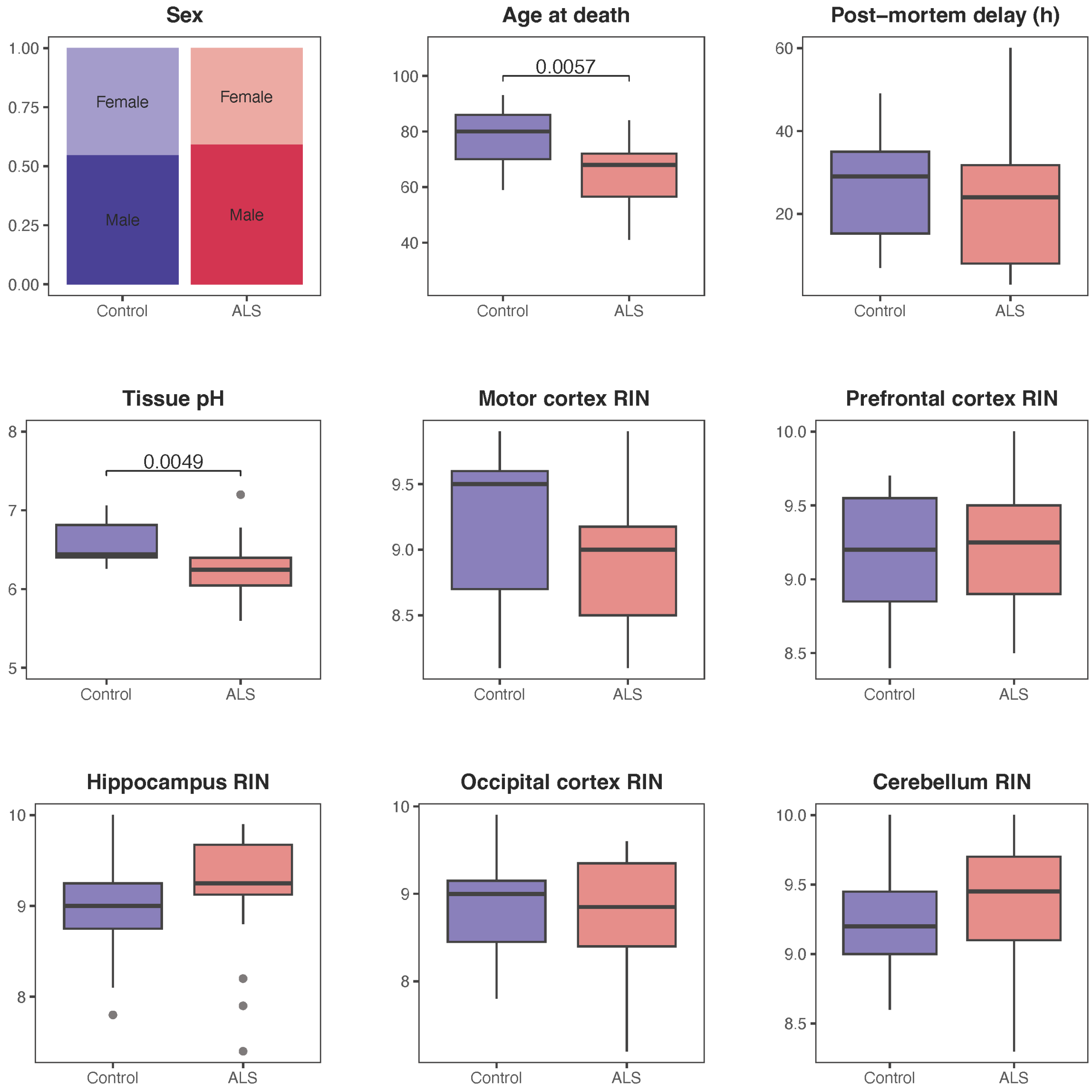

Fig. S3: Comparison of key samples features between ALS patients (n=22) and controls (n=11). Age at death and cerebellar tissue pH were significantly different between groups (Welch Two Sample t-test p-value is displayed; significance threshold of p-value < 0.05). Complete results of statistical comparisons can be found in Table S4.

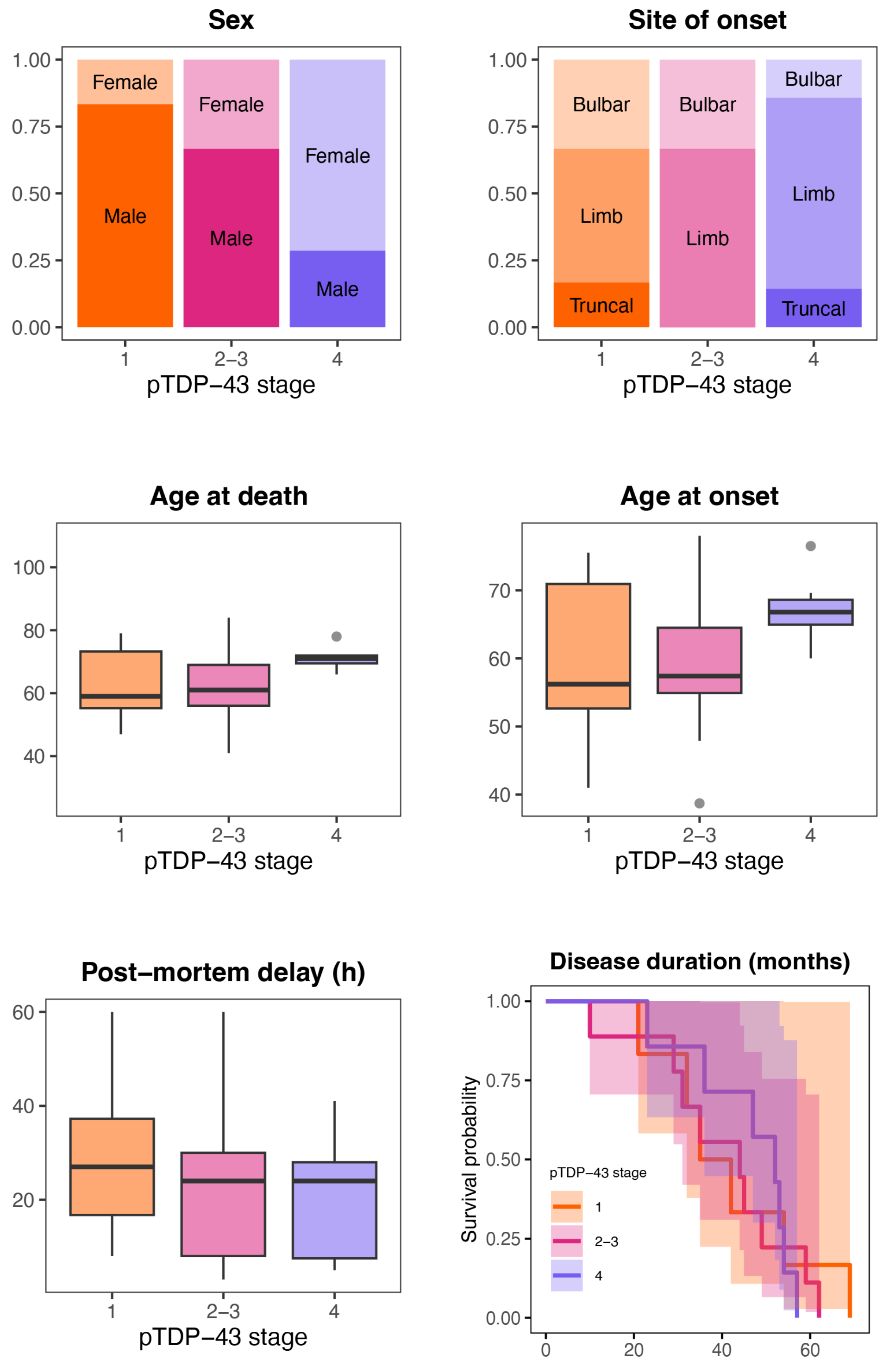

Fig. S4: Comparison of clinical features between ALS patient pTDP-43 pathology stage groups. All ALS patients were were sub-classified by their post-mortem pTDP-43 pathology stage (6 stage 1, 9 stage 2-3, 7 stage 4). No features were significantly different between pTDP-43 pathology stage groups (significance threshold of p-value < 0.05). Complete results of statistical comparisons can be found in Table S5.

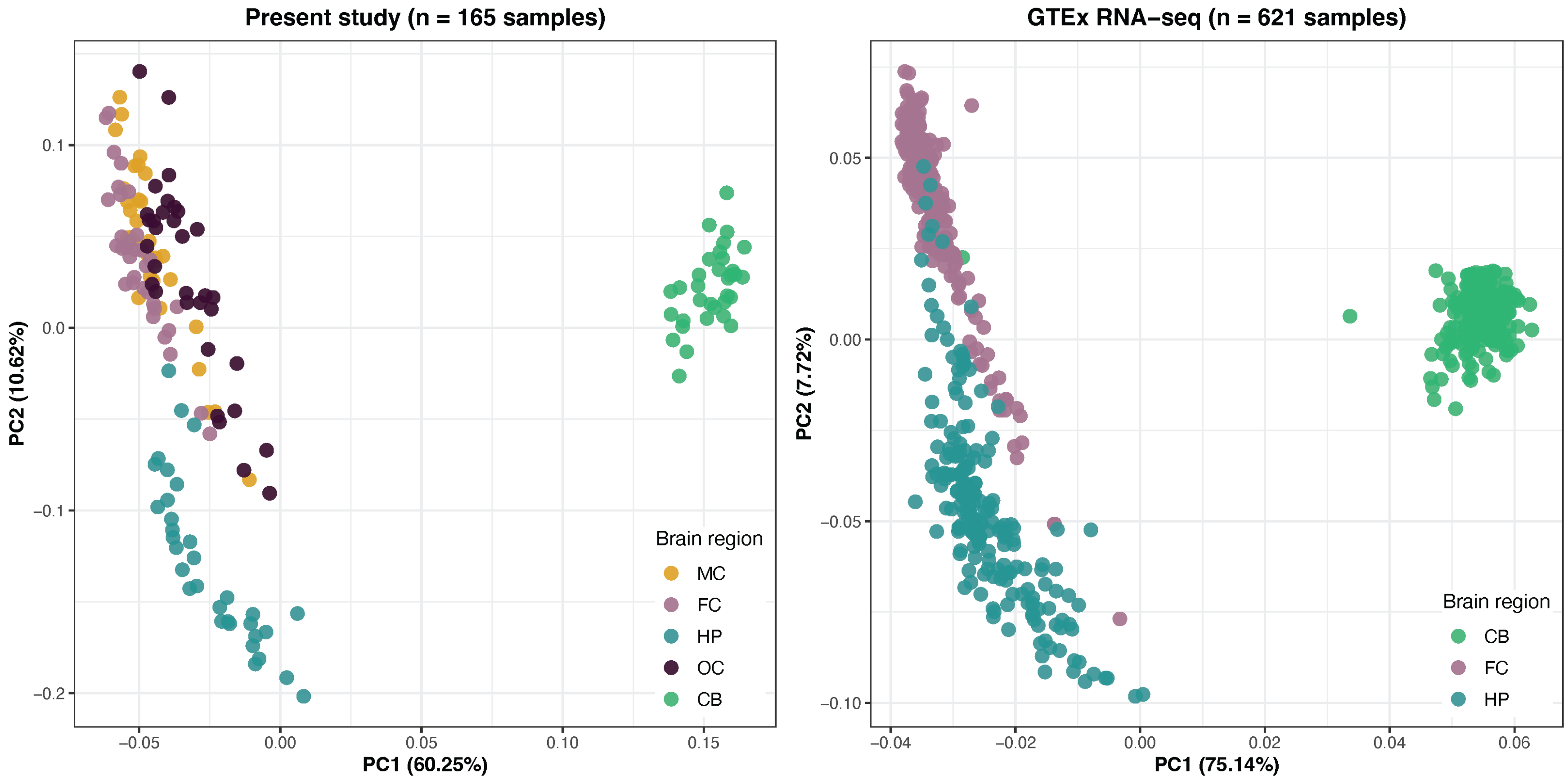

Fig. S5: Comparison of the current study's brain gene expression distribution to the Genotype Tissue Expression (GTEx) Consortium. Principal component analysis was performed on our 165 brain RNA-seq samples and 621 brain RNA-seq samples from GTEx (frontal_cortex_ba9, cerebellar_hemisphere and brain_hippocampus; 2017-06-05_v8). 500 genes showing the highest variance were considered for each data set. The distribution of brain region gene expression was visualised by 2D plotting of principal components. MC, motor cortex; FC, frontal cortex; HP, hippocampus; OC, occipital cortex; CB, cerebellum.

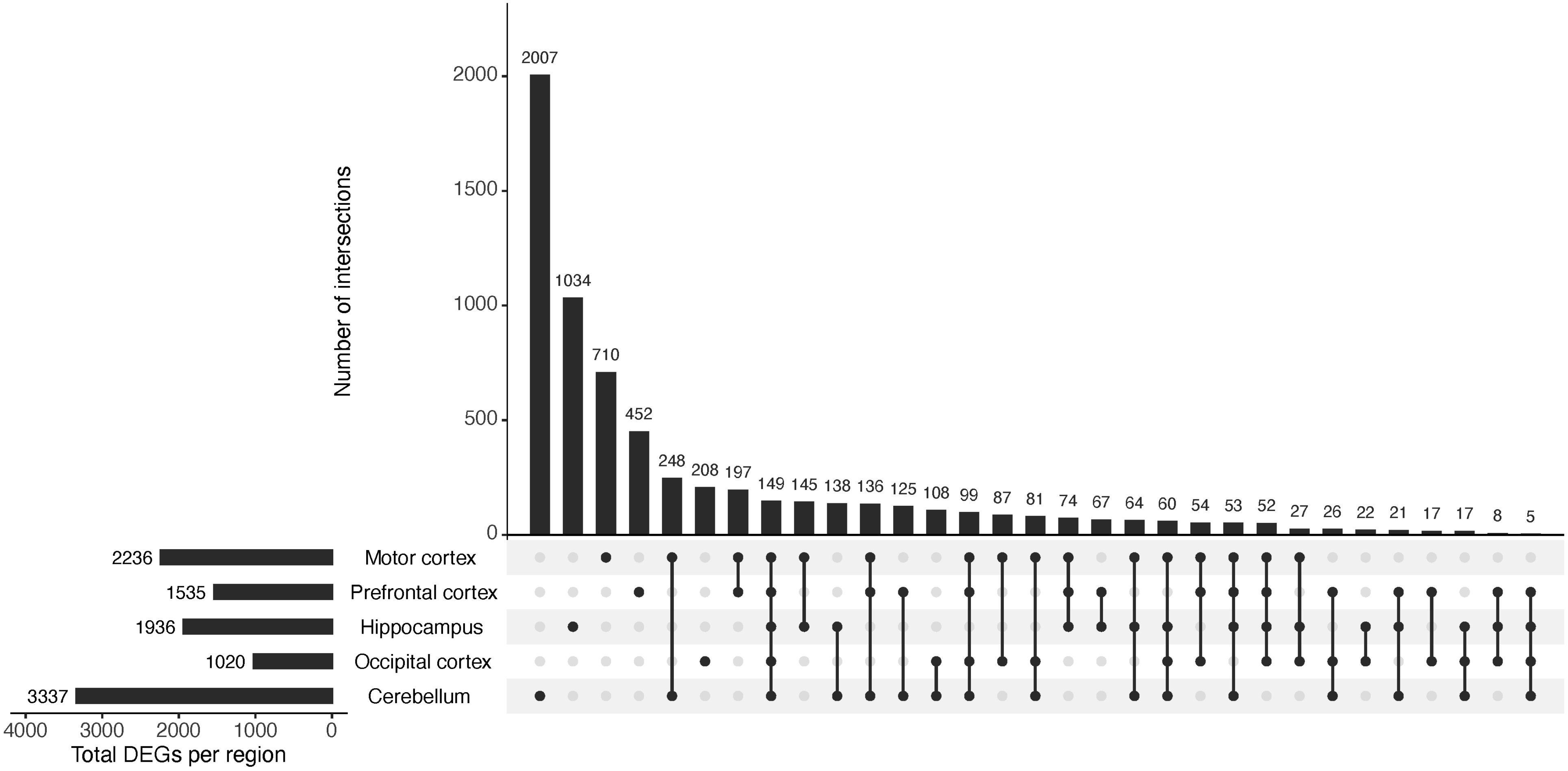

Fig. S6: Overlap of ALS-control differentially expressed genes between the five examined brain regions. In the upset plot the brain regions involved in each intersection are indicated by a filled dot. Significant differentially expressed genes were defined as those with FDR < 0.05. DEGs, differentially expressed genes.

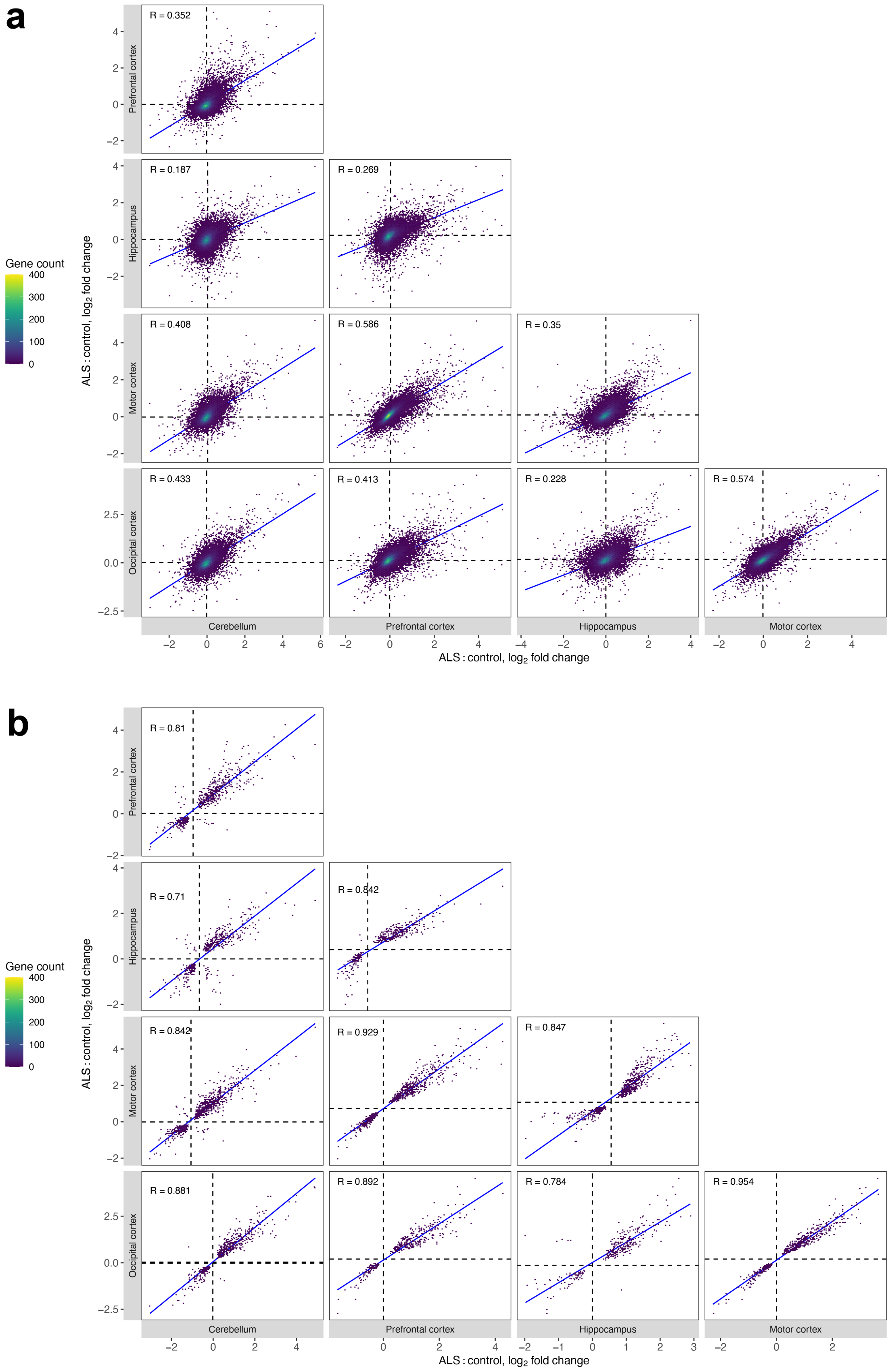

Fig. S7: Correlation of ALS:control $\log_2$ fold changes between each brain region. Each point represents a gene considering either **(a)** all tested genes or **(b)** common differentially expressed genes for a given region pair. Regression line is shown in blue with corresponding adjusted R^2 value shown at the top of each plot.

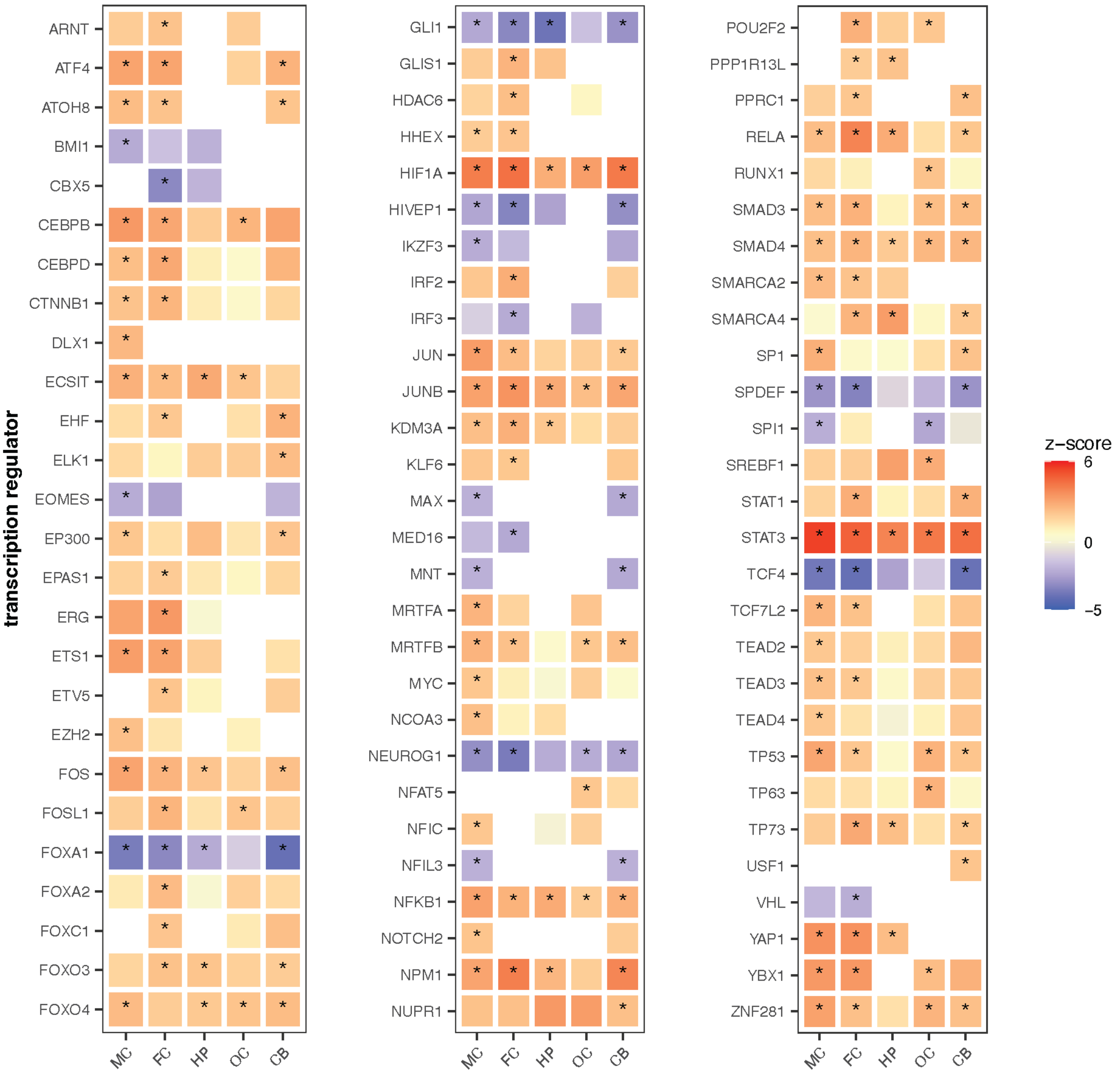

Fig. S8: Transcription regulators predicted to be activated or inhibited in ALS patients relative to controls. QIAGEN IPA upstream regulator analysis was performed on differential gene expression results using a cut-off of FDR < 0.05 and $|\log_2$ fold change$| > 0.263$. Transcription regulators were filtered for those with an $|$activation z-score$| \geq 2$ and p-value of overlap < 0.05 in at least one brain region. Tiles are coloured according to activation z-score. No z-score was calculated when there were insufficient differentially expressed genes downstream of the transcriptional regulator to make a prediction. Asterix indicates transcriptional regulators considered to be significantly activated (z-score $\geq$ 2, p-value of overlap < 0.05) or inhibited (z-score $\leq$ -2, p-value of overlap < 0.05).

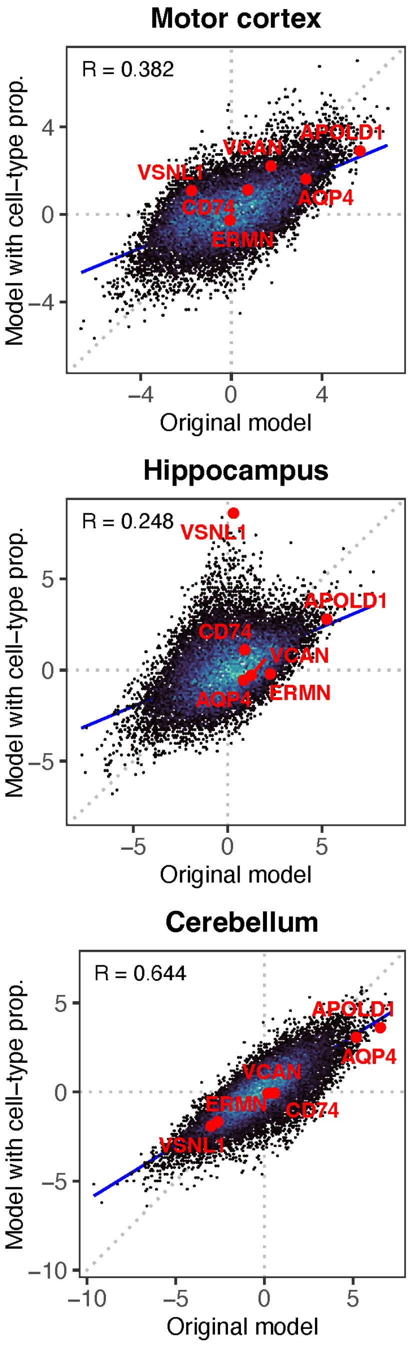

Fig. S9: Comparison of per-gene *t* statistic values when estimated cell-type proportions are added to ALS-control differential gene expression models. Estimated proportions for six cell types were obtained using dtangle and human cortical scRNA-seq as a reference (Darmanis et al., 2015). Astrocyte, endothelial, neuron, oligodendrocyte and oligodendrocyte progenitor cell proportions were added as additional covariates to the original models (presented in Table 2) for limma differential gene expression analysis. Microglia estimated proportions were excluded as the nature of cell-type proportions (i.e. sums to one) makes its inclusion redundant. Resulting *t* statistic values were correlated, with the regression line shown in blue and corresponding adjusted R^2 value shown at the top of each plot. Point colour indicates the density of genes. The dtangle top-ranked marker gene for each cell type is shown in red (*AQP4* for astrocytes, *APOLD1* for endothelial cells, *CD74* for microglia, *VSNL1* for neurons, *ERMN* for oligodendrocytes, *VCAN* for oligodendrocyte progenitor cells).

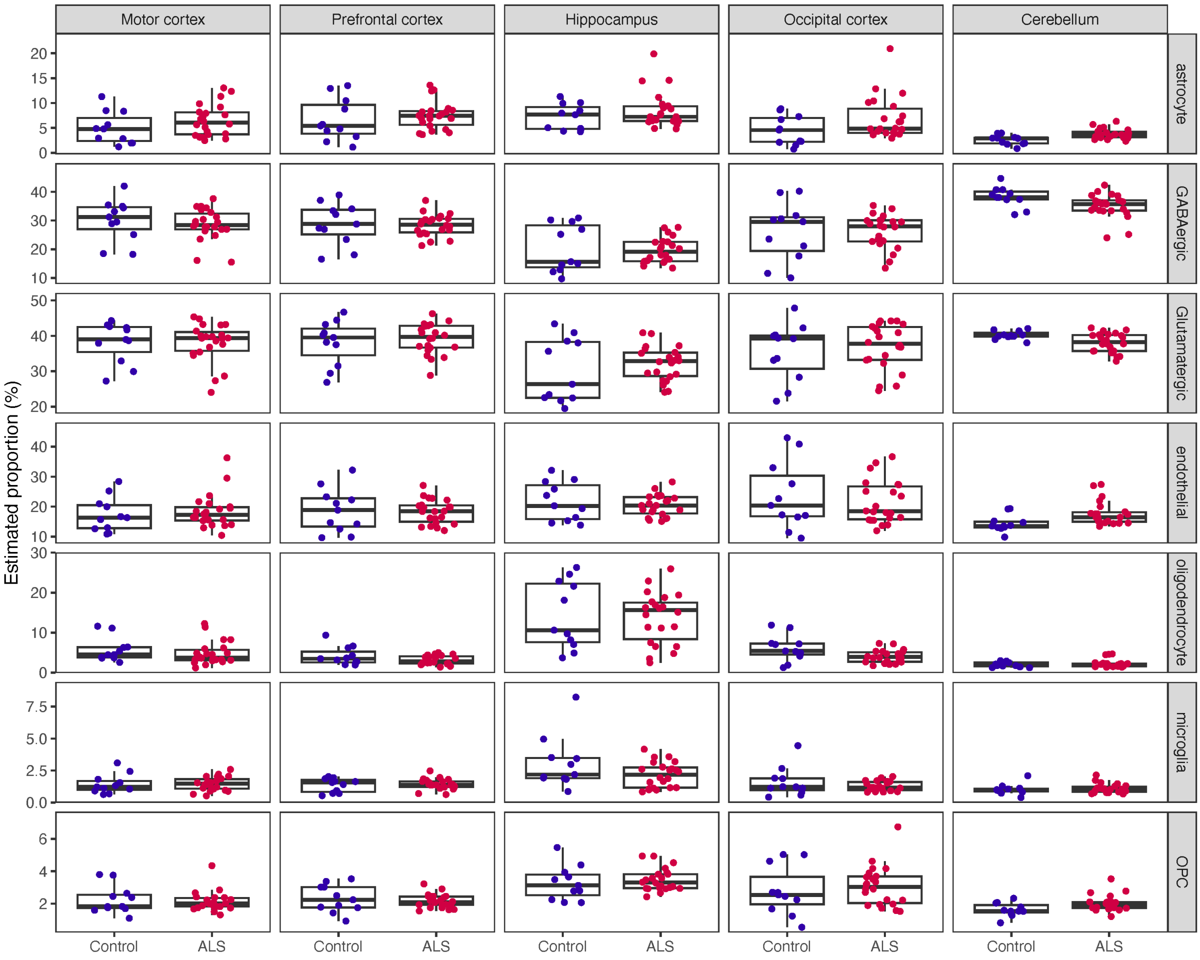

Fig. S10: Estimated proportions of seven major cell types in ALS patients versus controls across all five brain regions. Cell-type deconvolution was performed on all 165 brain RNA-seq samples using dtangle. Here, cortex-derived single-nucleus RNA-seq from the Allen Brain Institute was used as the reference data. To test for ALS-control differences in cell type proportions, a linear model (proportion $\sim$ disease_status + age) was fitted for each brain region and cell-type combination, and a Bonferroni correction was applied to disease_status p-values. No cell-type proportions were significantly different between ALS patients and controls (significance threshold of adjusted p-value < 0.05). OPC, oligodendrocyte progenitor cell.

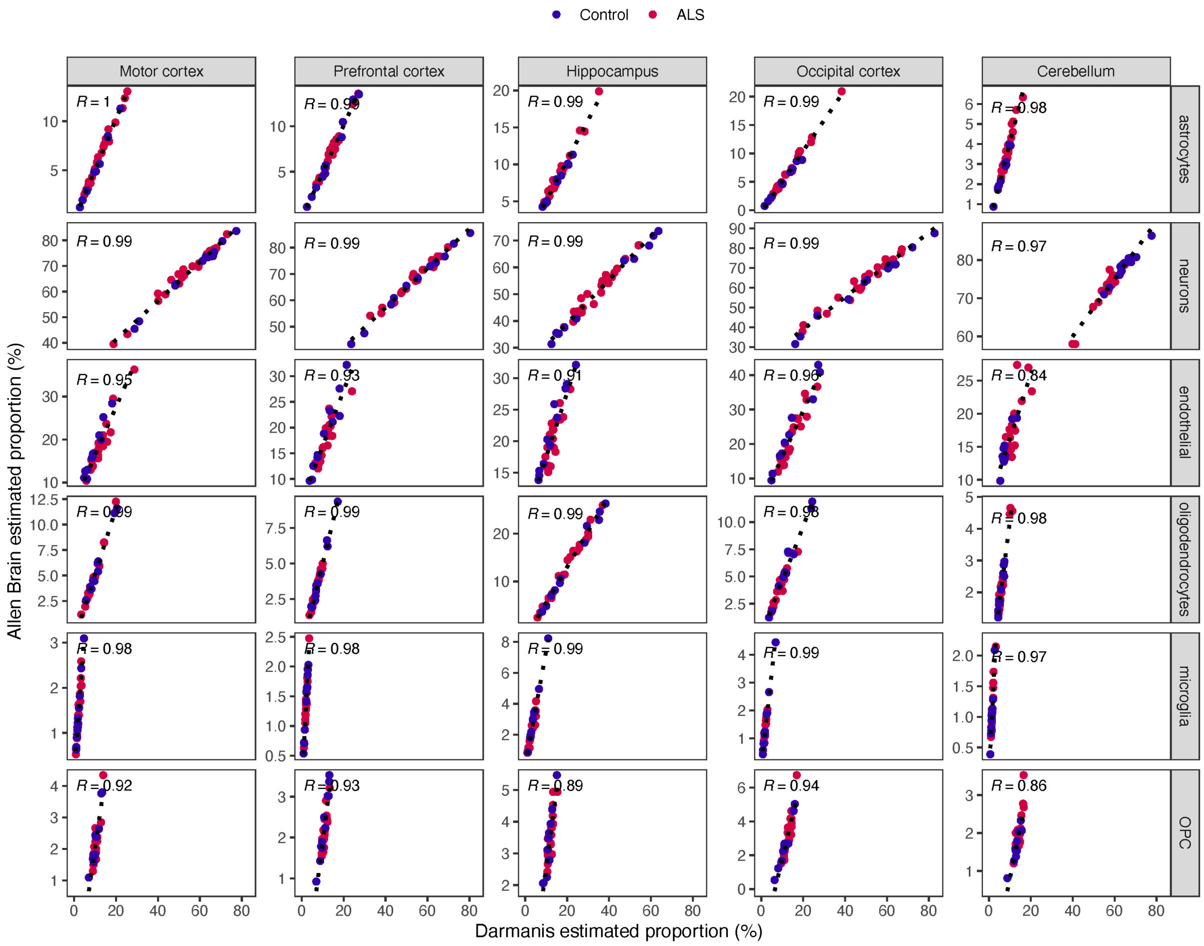

Fig. S11: Correlation of dtangle estimated proportions of six major cell types for Darmanis versus Allen Brain Institute reference data. Darmanis scRNA-seq and Allen Brain Institute snRNA-seq were both derived from human cortex tissue. Estimated proportions of GABAergic and glutamatergic cells, obtained when using the Allen Brain Institute reference data, were summed under neurons. OPC, oligodendrocyte progenitor cell.

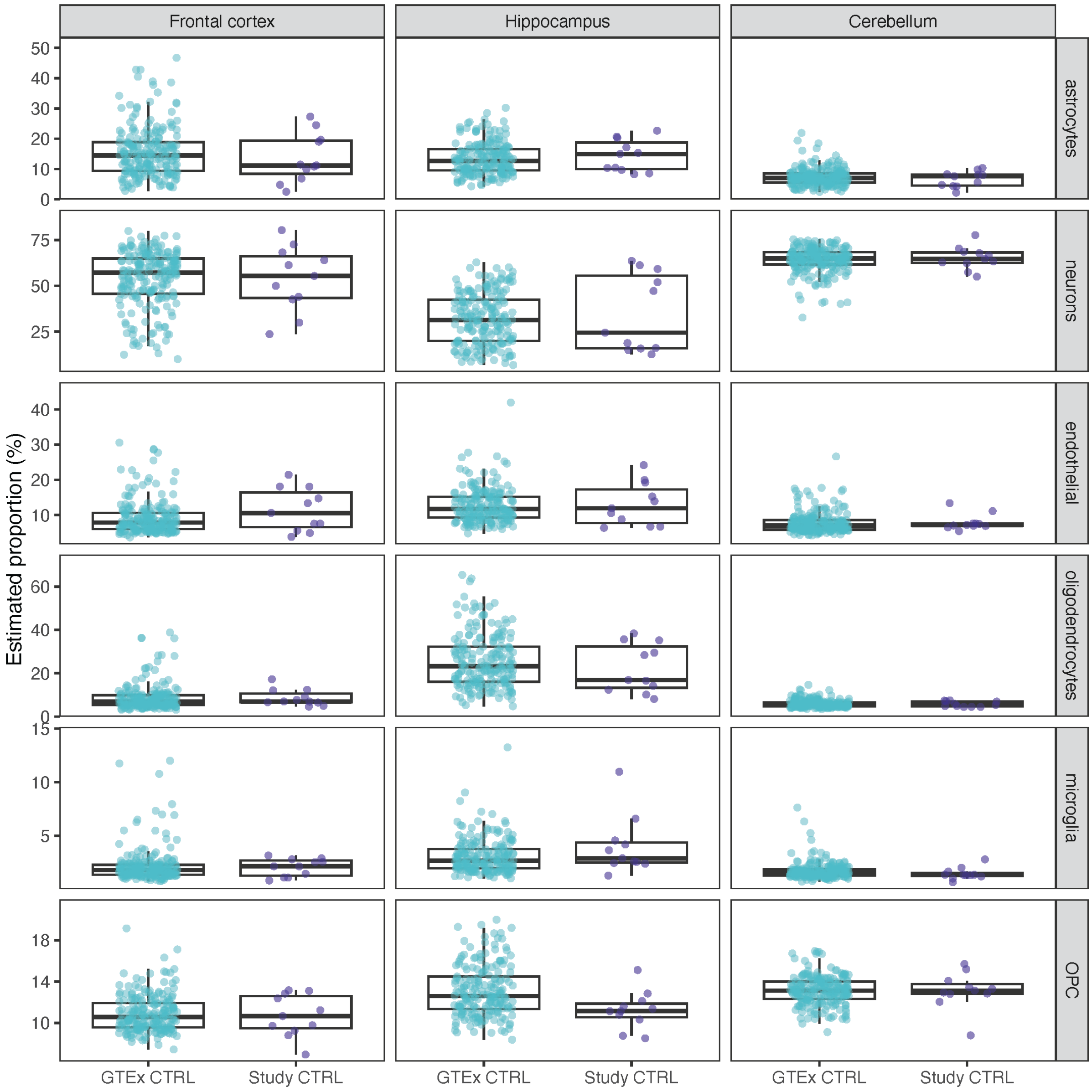

Fig. S12: Estimated cell-type proportions for Genotype Tissue Expression (GTEx) Consortium brain samples versus controls. Cell-type deconvolution was performed on 621 brain RNA-seq samples from GTEx using dtangle: frontal_cortex_ba9 (n=209), cerebellar_hemisphere (n=215) and brain_hippocampus (n=197)(2017-06-05_v8). Cortex-derived single cell RNA-seq (Darmanis et al., 2015) was used as the reference data. To test for differences in cell type proportions between GTEx samples (GTEx CTRL) and control samples from the present study (Study CTRL), a Wilcoxon signed-rank test was performed with Bonferroni correction of p-values. No cell-type proportions were significantly different between GTEx CTRL and Study CTRL (significance threshold of adjusted p-value < 0.05). OPC, oligodendrocyte progenitor cell.

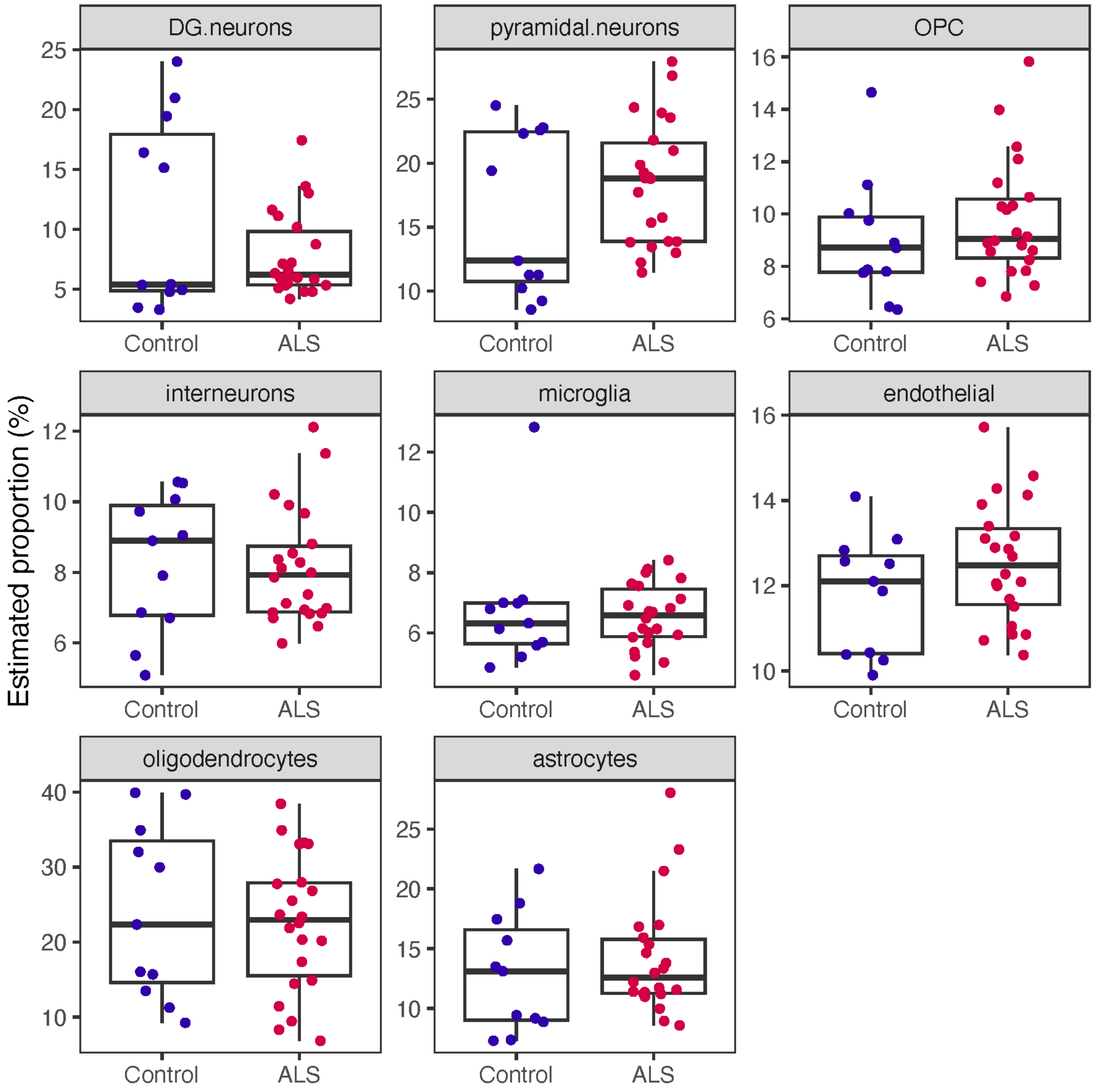

Fig. S13: Estimated proportions of eight hippocampus cell types in ALS patients versus controls. Cell-type deconvolution was performed using dtangle on all 33 hippocampus RNA-seq samples. Hippocampus single-nucleus RNA-seq (Ayhan et al., 2021) was used as the reference data. To test for ALS-control differences in cell type proportions, a linear model (proportion $\sim$ disease_status + age) was fitted for each cell-type, and a Bonferroni correction was applied to disease_status p-values. No cell-type proportions were significantly different between ALS patients and controls (significance threshold of adjusted p-value < 0.05). DG, dentate gyrus; OPC, oligodendrocyte progenitor cell.

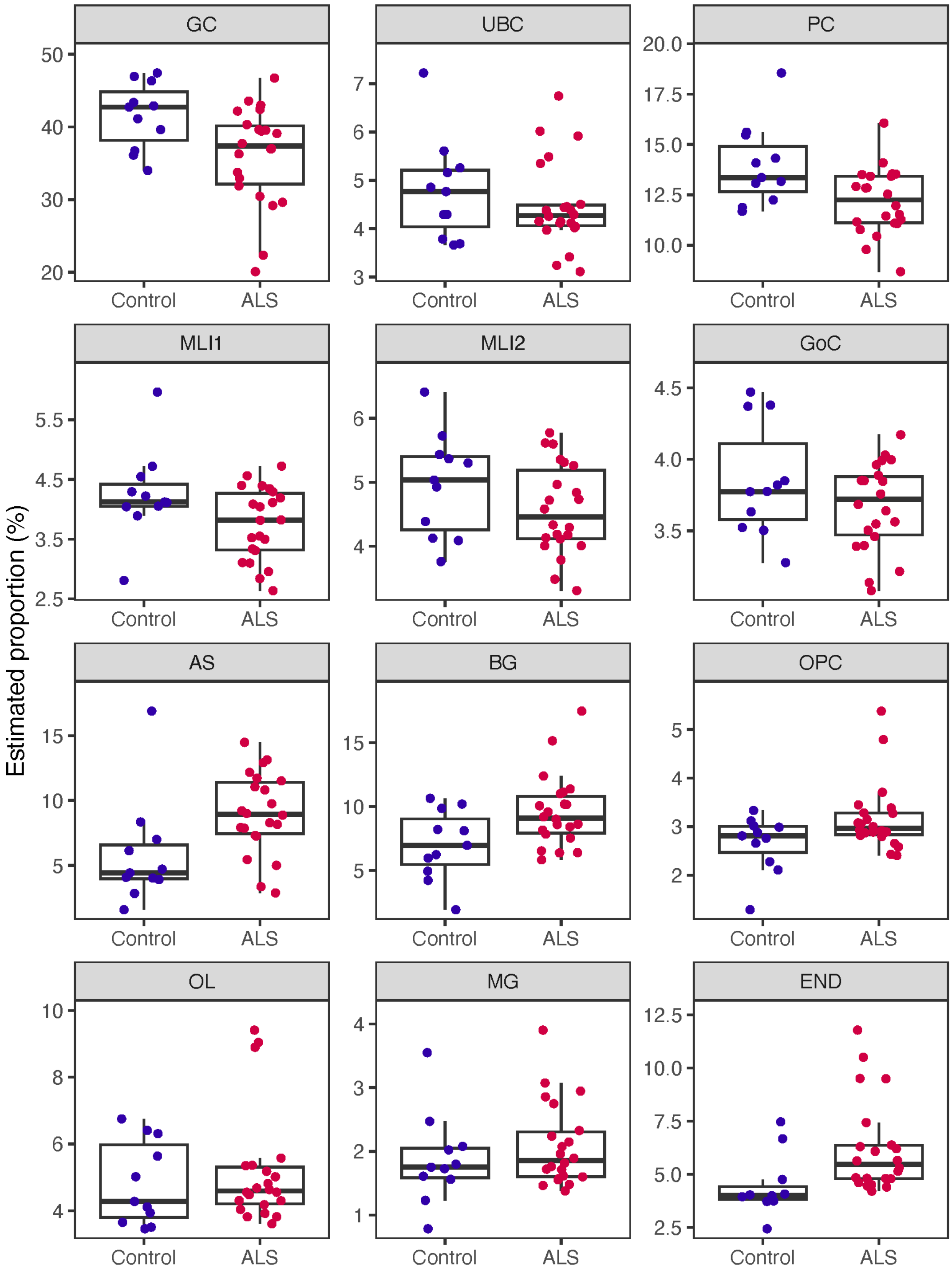

Fig. S14: Estimated proportions of 12 cerebellum cell types in ALS patients versus controls. Cell-type deconvolution was performed using dtangle on all 33 cerebellum RNA-seq samples. Cerebellum single-nucleus RNA-seq (Tejwani et al., 2024) was used as the reference data. To test for ALS-control differences in cell type proportions, a linear model (proportion $\sim$ disease_status + age) was fitted for each cell-type, and a Bonferroni correction was applied to disease_status p-values. No cell-type proportions were significantly different between ALS patients and controls (significance threshold of adjusted p-value < 0.05). GC, granule cells; UBC, unipolar brush cells; PC, Purkinje cells; MLI1, molecular layer interneuron population 1; MLI2, molecular layer interneuron population 2; GoC, Golgi cells; AS, astrocytes; BG, Bergmann glia; OPC, oligodendrocyte progenitor cells; OL, oligodendrocytes; MG, microglia; END, endothelial cells.

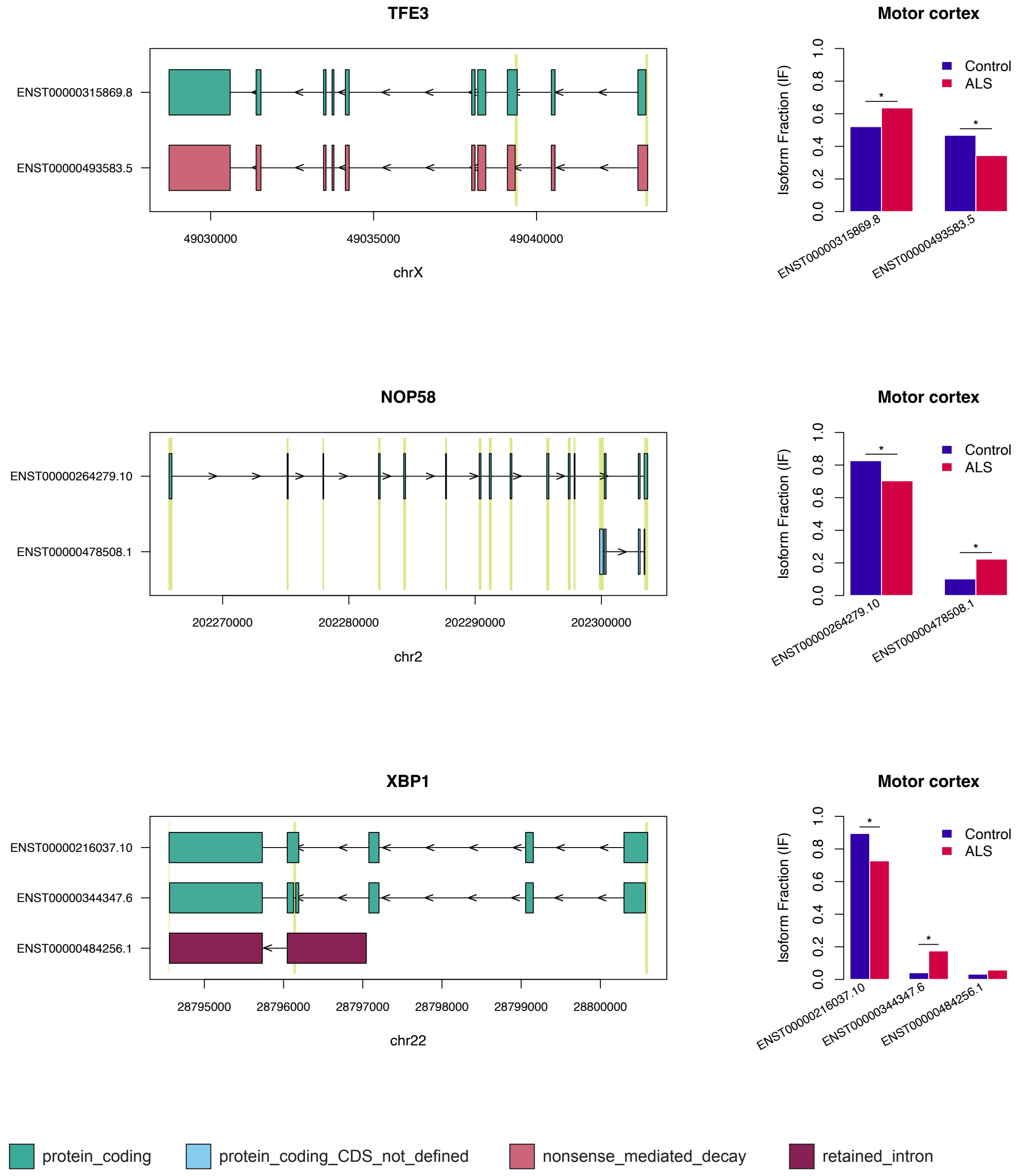

Fig. S15: Visual of 12 genes identified to have differential transcript usage of two isoforms between ALS patients and controls. *Left panels*: Visual depiction of transcripts that contributed $\geq 5\%$ of total gene expression. Exons are shown as boxes and introns as lines. Chromosome position is shown along the x axis and the strand is indicated by arrow heads where ">" indicates forward strand and "<" indicates reverse strand. Transcript colour indicates the type of transcript as indicated in the bottom key. The yellow shading behind transcripts highlights the exonic regions that differ between the two significant differentially used transcripts. *Right panels*: Comparison of isoform fractions between ALS patients and controls. Significant differences (q value < 0.05) are indicated by an asterisk.

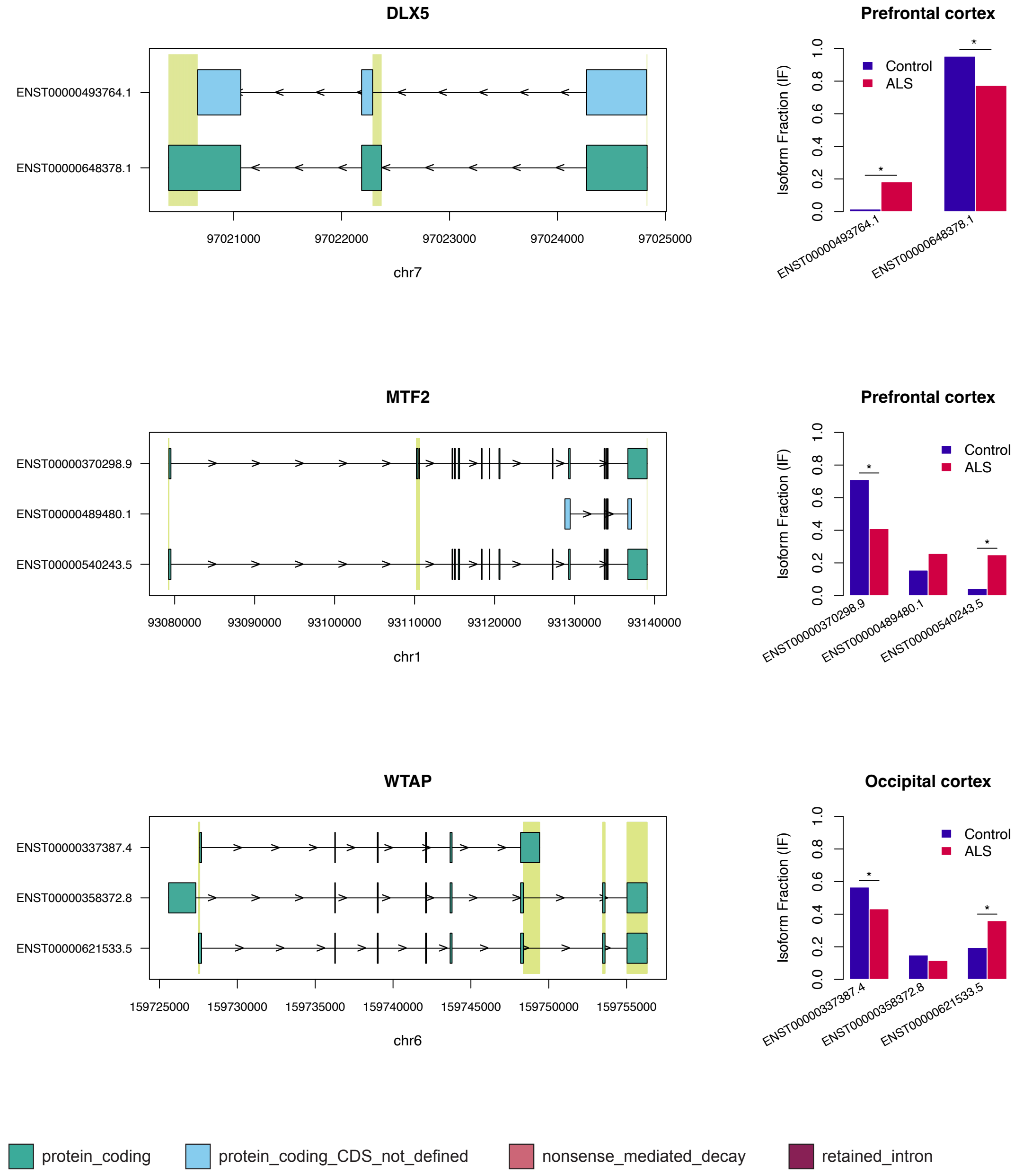

Fig. S15 (continued): Visual of 12 genes identified to have differential transcript usage of two isoforms between ALS patients and controls. *Left panels*: Visual depiction of transcripts that contributed $\geq 5\%$ of total gene expression. Exons are shown as boxes and introns as lines. Chromosome position is shown along the x axis and the strand is indicated by arrow heads where “>” indicates forward strand and “<” indicates reverse strand. Transcript colour indicates the type of transcript as indicated in the bottom key. The yellow shading behind transcripts highlights the exonic regions that differ between the two significant differentially used transcripts. *Right panels*: Comparison of isoform fractions between ALS patients and controls. Significant differences (q value < 0.05) are indicated by an asterisk.

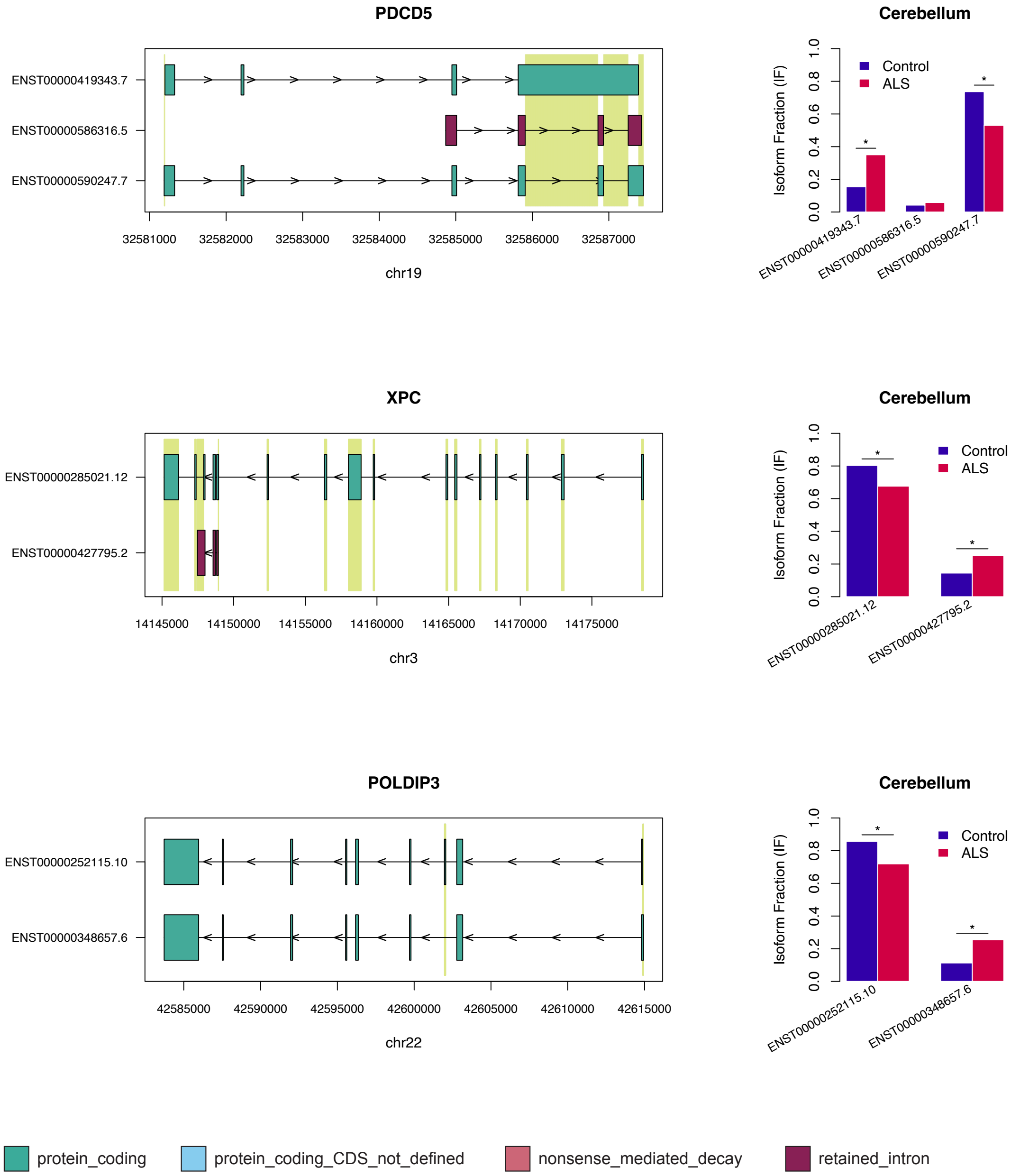

Fig. S15 (continued): Visual of 12 genes identified to have differential transcript usage of two isoforms between ALS patients and controls. *Left panels*: Visual depiction of transcripts that contributed $\geq 5\%$ of total gene expression. Exons are shown as boxes and introns as lines. Chromosome position is shown along the x axis and the strand is indicated by arrow heads where "$>$" indicates forward strand and "$<$" indicates reverse strand. Transcript colour indicates the type of transcript as indicated in the bottom key. The yellow shading behind transcripts highlights the exonic regions that differ between the two significant differentially used transcripts. *Right panels*: Comparison of isoform fractions between ALS patients and controls. Significant differences (q value $<$ 0.05) are indicated by an asterisk.

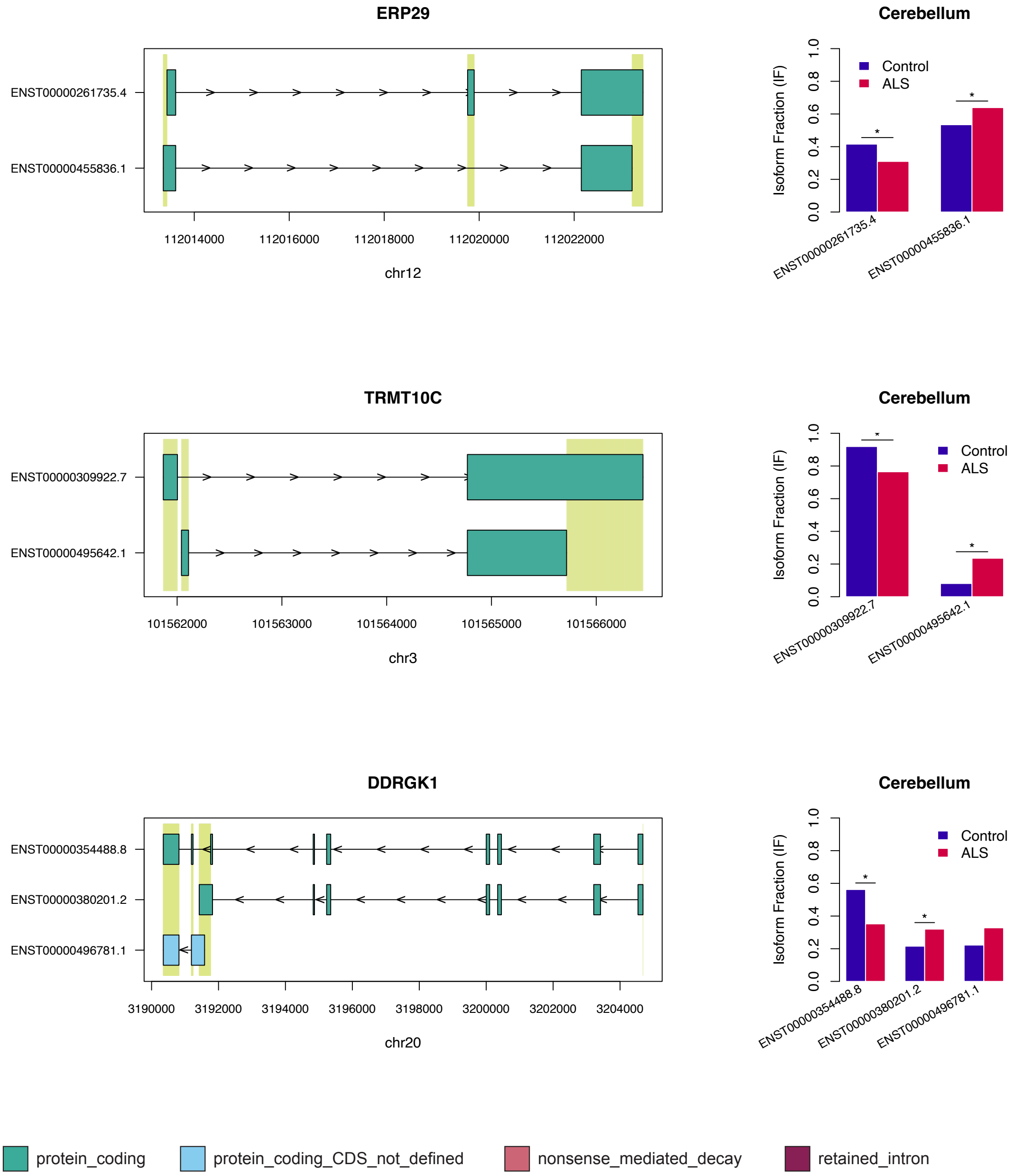

Fig. S15 (continued): Visual of 12 genes identified to have differential transcript usage of two isoforms between ALS patients and controls. *Left panels*: Visual depiction of transcripts that contributed $\geq 5\%$ of total gene expression. Exons are shown as boxes and introns as lines. Chromosome position is shown along the x axis and the strand is indicated by arrow heads where “>” indicates forward strand and “<” indicates reverse strand. Transcript colour indicates the type of transcript as indicated in the bottom key. The yellow shading behind transcripts highlights the exonic regions that differ between the two significant differentially used transcripts. *Right panels*: Comparison of isoform fractions between ALS patients and controls. Significant differences (q value $<$ 0.05) are indicated by an asterisk.

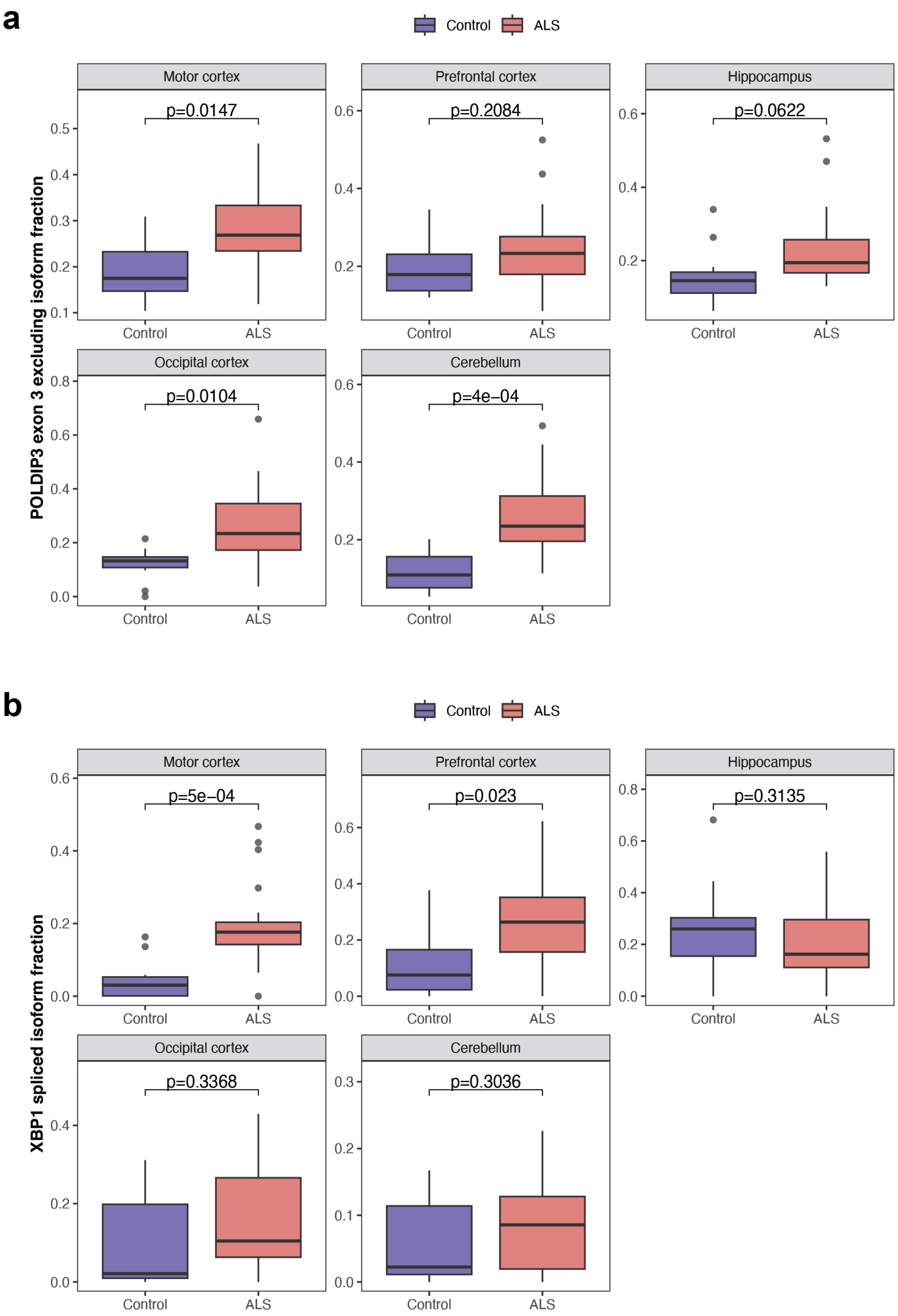

Fig. S16: Simplified isoform fraction of *POLDIP3* and *XBP1* differentially used transcripts. For each gene two transcripts were identified as significantly differentially used in opposing directions (FDR < 0.05 and $\geq 10\%$ change in isoform usage) between ALS patients and controls. A simplified isoform fraction was calculated considering only the two differentially used transcripts (i.e. isoform 1 / [isoform 1 + isoform 2]) to see if their relative usage was altered in other brain regions. **(a)** For *POLDIP3*, isoform 1 is ENST00000348657.6 (exon 3 excluding) and isoform 2 is ENST00000252115.10 (canonical). **b** For *XBP1*, isoform 1 is ENST00000344347.6 ("spliced") and isoform 2 is ENST00000216037.10 ("unspliced"). A linear model (isoform fraction $\sim$ disease_status + age) was fitted and p-values for disease_status are displayed.

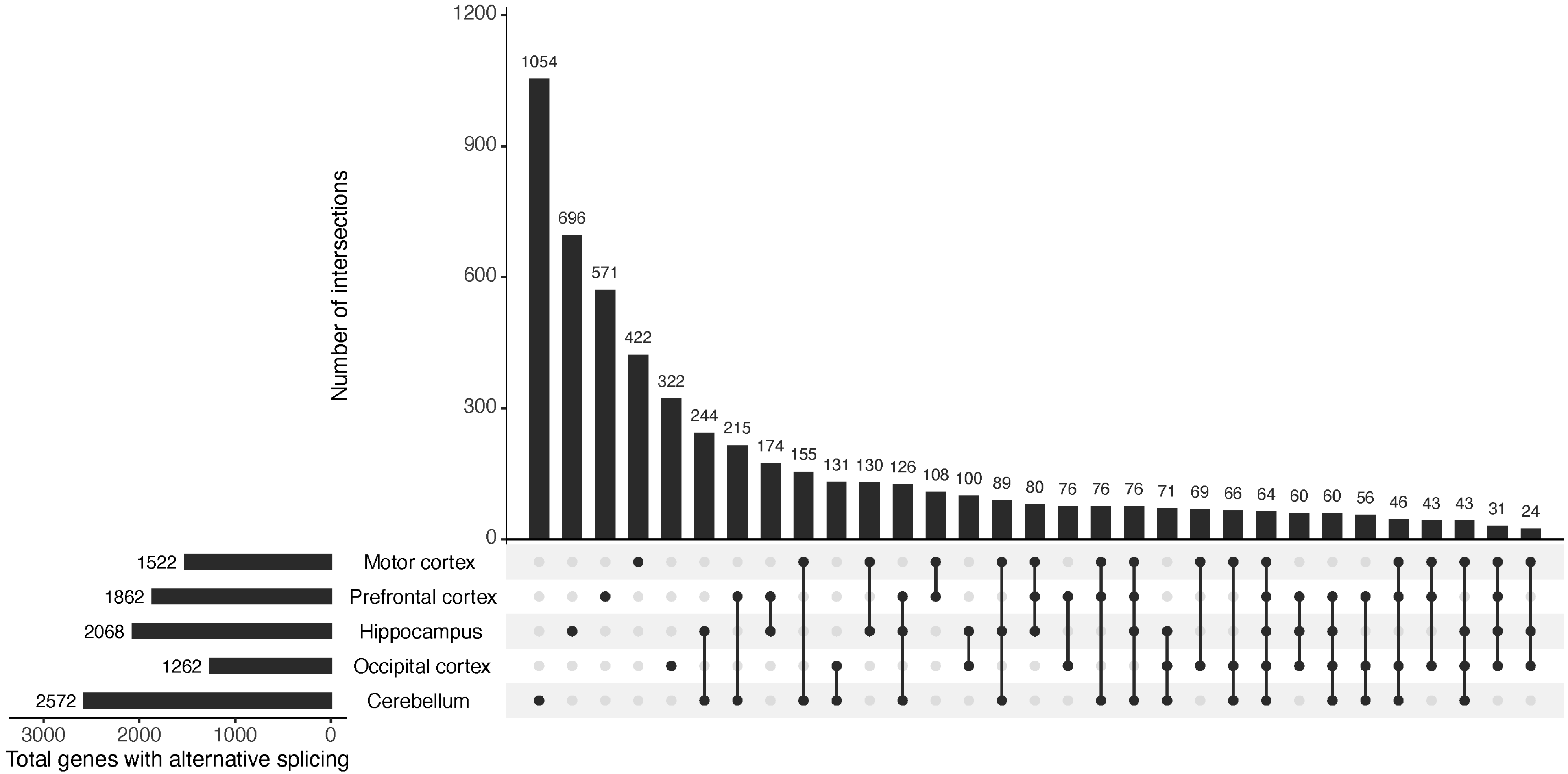

Fig. S17: Overlap of ALS-control alternatively spliced genes between the five examined brain regions as determined by MAJIQ. In the upset plot the brain regions involved in each intersection are indicated by a filled dot. Significant alternative splicing events were defined as those involving a junction with $|\Delta\Psi| > 10\%$ and Wilcoxon p-value < 0.05.

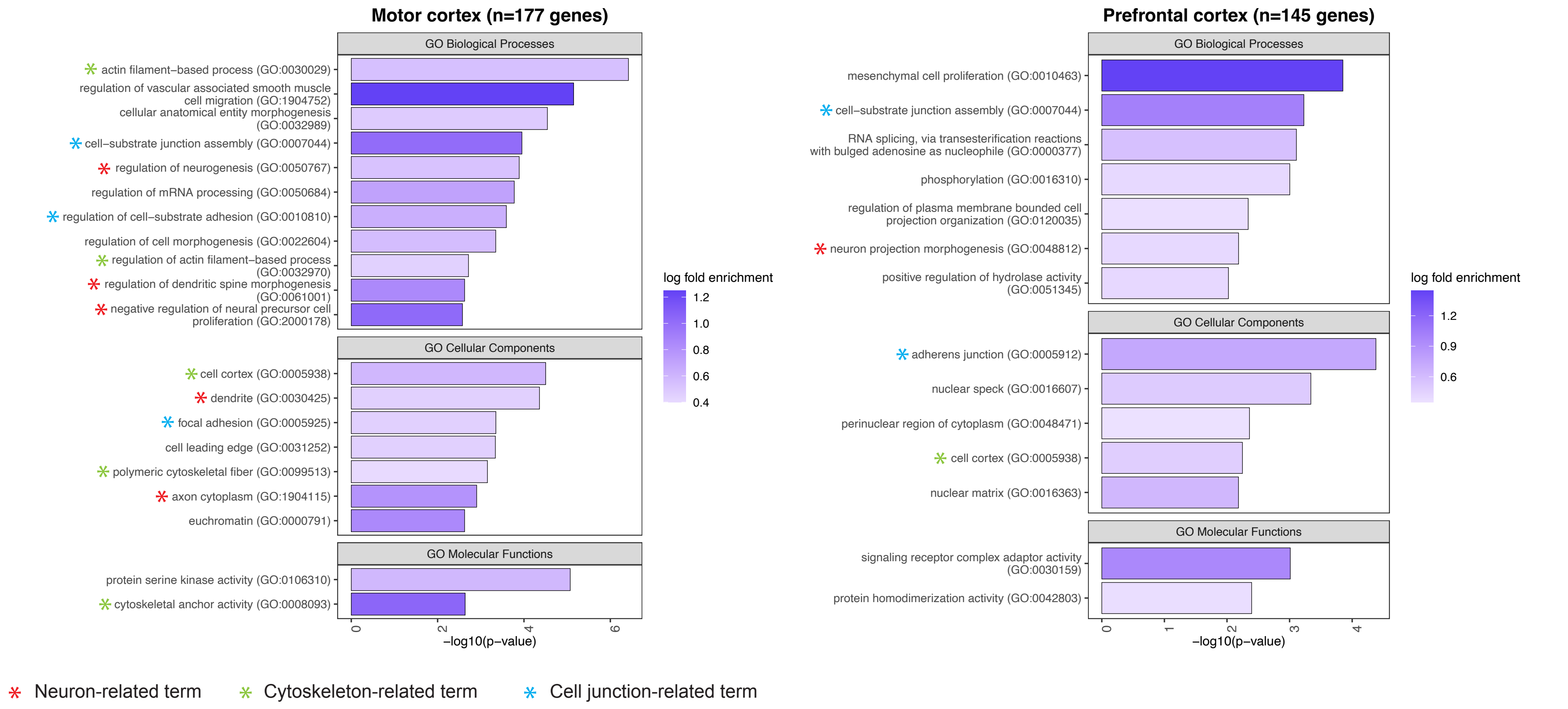

Fig. S18: Gene ontology (GO) enrichment analysis result for genes identified as differentially spliced between ALS patients and controls. The overlap of genes identified by both MAJIQ ($|\Delta\Psi| > 10\%$, Wilcoxon p-value < 0.05) and LeafCutter (FDR < 0.05) per brain region, was used for Metascape enrichment analysis. The number of overlapping differentially spliced genes between the two tools is listed at the top of each plot. The most representative member (lowest p-value) of each GO term cluster is displayed. Log2 fold change indicates enrichment of member genes in differentially spliced genes versus all genes quantified by MAJIQ. Coloured asterisks highlight recurring categories.

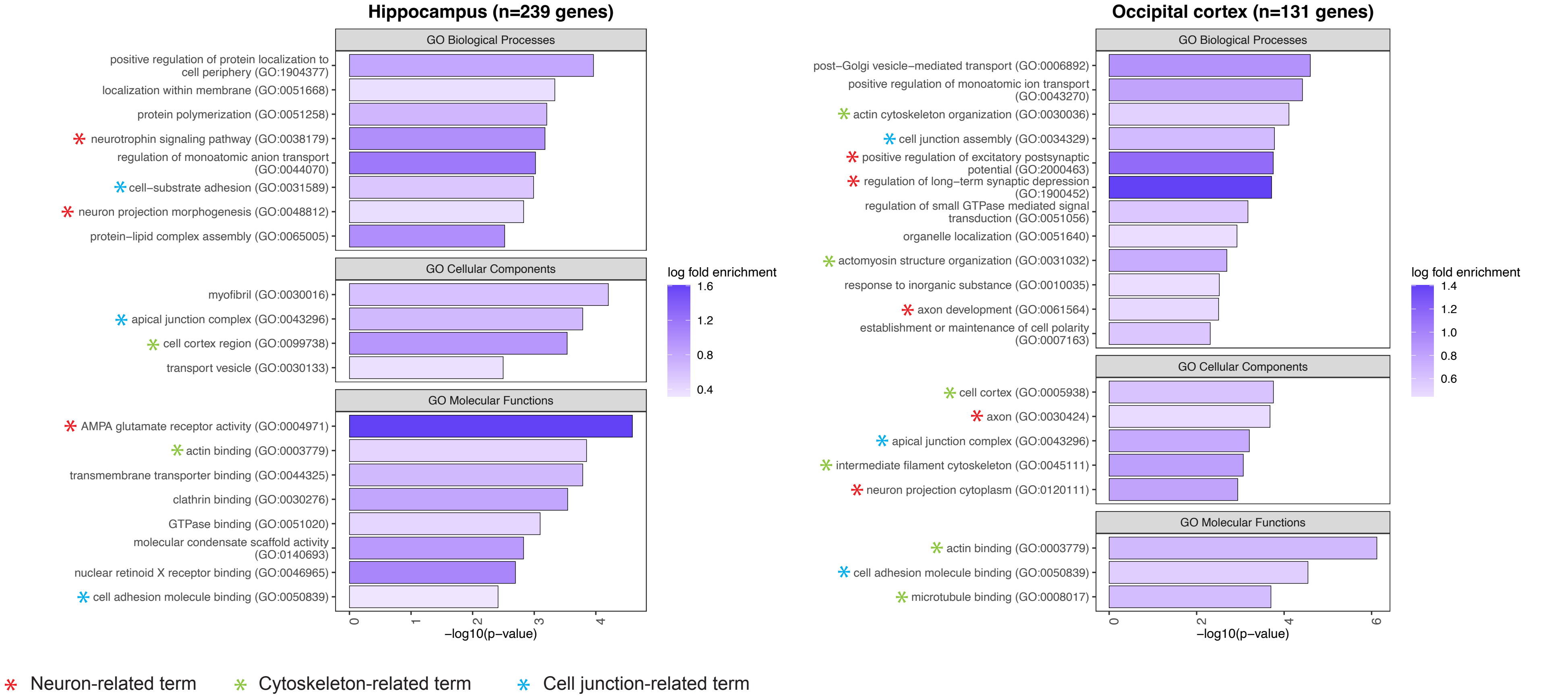

Fig. S18 (continued): Gene ontology (GO) enrichment analysis result for genes identified as differentially spliced between ALS patients and controls. The overlap of genes identified by both MAJIQ ($|\Delta\Psi| > 10\%$, Wilcoxon p-value < 0.05) and LeafCutter (FDR < 0.05) per brain region, was used for Metascape enrichment analysis. The number of overlapping differentially spliced genes between the two tools is listed at the top of each plot. The most representative member (lowest p-value) of each GO term cluster is displayed. Log2 fold change indicates enrichment of member genes in differentially spliced genes versus all genes quantified by MAJIQ. Coloured asterisks highlight recurring categories.

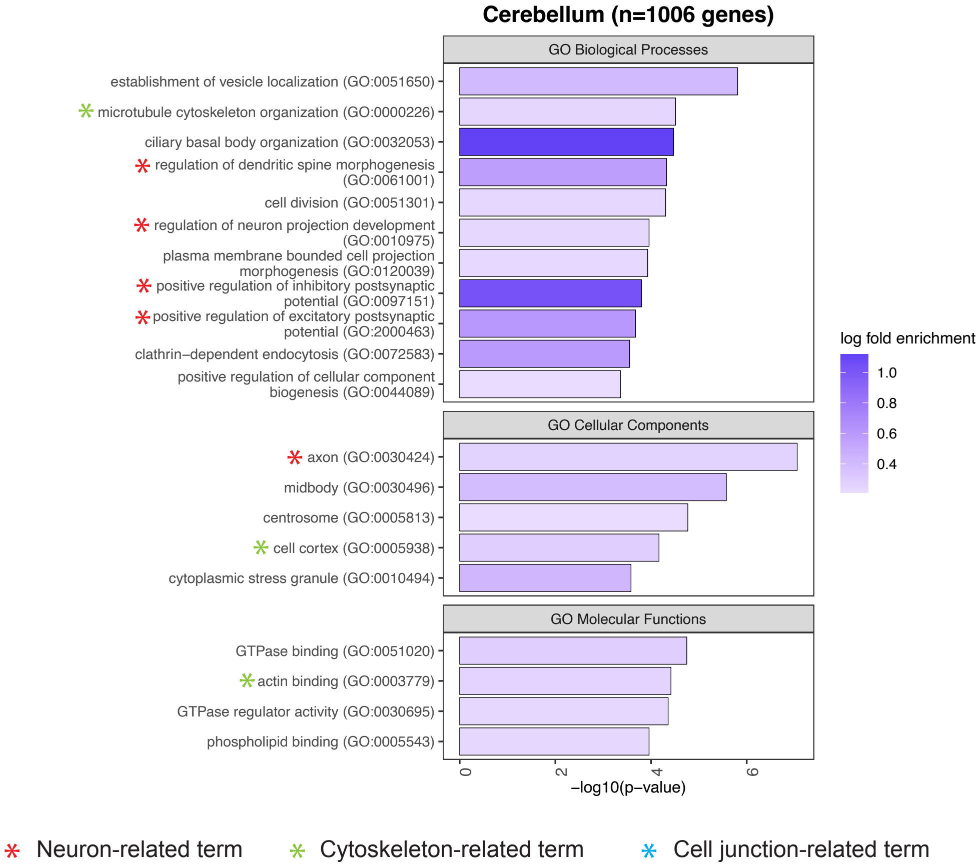

Fig. S18 (continued): Gene ontology (GO) enrichment analysis result for genes identified as differentially spliced between ALS patients and controls. The overlap of genes identified by both MAJIQ ($|\Delta\Psi| > 10\%$, Wilcoxon p-value < 0.05) and LeafCutter (FDR < 0.05) per brain region, was used for Metascape enrichment analysis. The number of overlapping differentially spliced genes between the two tools is listed at the top of each plot. The most representative member (lowest p-value) of each GO term cluster is displayed. Log2 fold change indicates enrichment of member genes in differentially spliced genes versus all genes quantified by MAJIQ. Coloured asterisks highlight recurring categories.

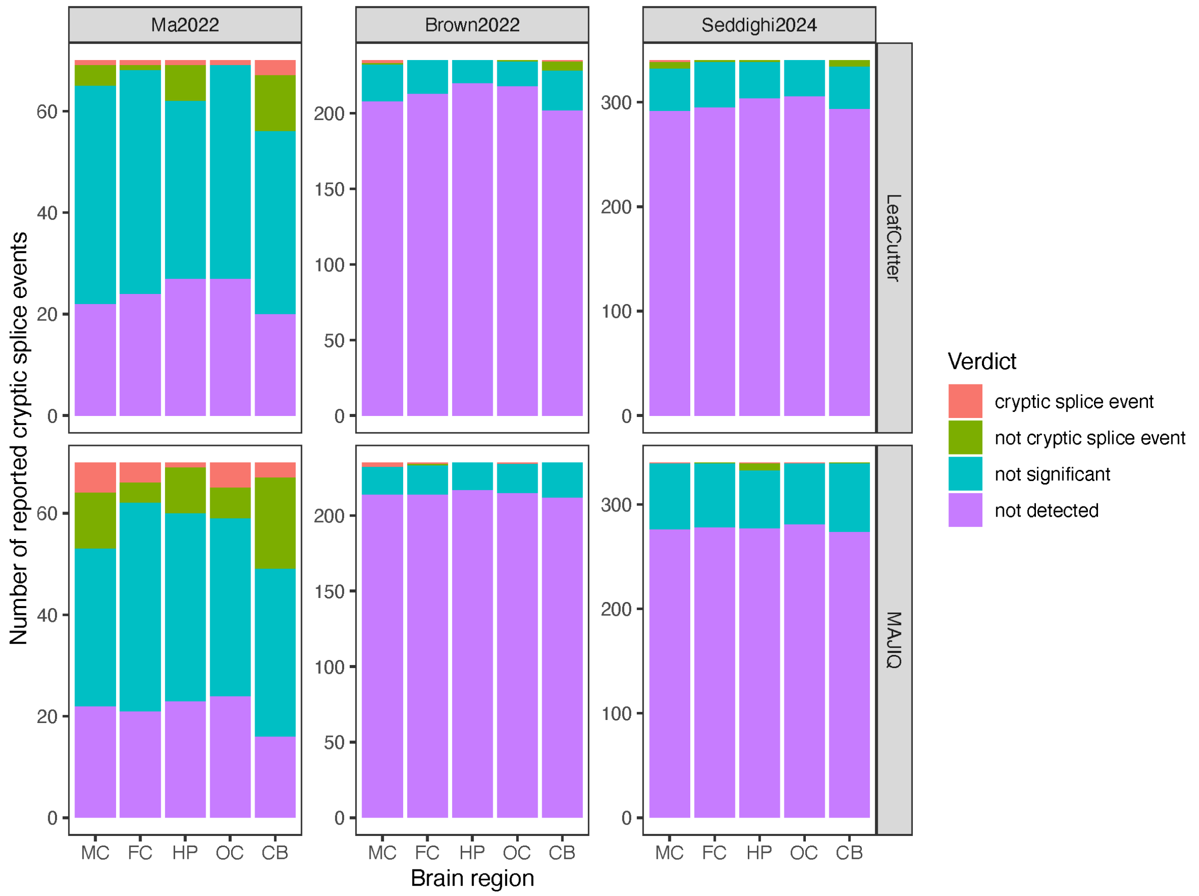

Fig. S19: Detection of literature-reported cryptic splicing events in bulk brain RNA-seq data. Verdict indicates the level of cryptic splice event detection: (1) "cryptic splice event" indicates that event met MAJIQ ($\Delta\Psi > 1\%$, Wilcoxon p-value < 0.05, de_novo_junction = 1) or LeafCutter ($\Delta\Psi > 1\%$, FDR < 0.05, classified as unannotated) definitions of a cryptic splice event; (2) "not cryptic splice event" indicates that the event was significant for MAJIQ ($|\Delta\Psi| > 1\%$, Wilcoxon p-value < 0.05) or LeafCutter (FDR < 0.05) but was not cryptic; (3) "not significant" indicates that the event coordinates were present in MAJIQ or LeafCutter output but did not meet significant threshold; (4) "not detected" indicates that the event coordinates were not present in MAJIQ or LeafCutter output. MC, motor cortex; FC, prefrontal cortex; HP, hippocampus; OC, occipital cortex; CB, cerebellum.

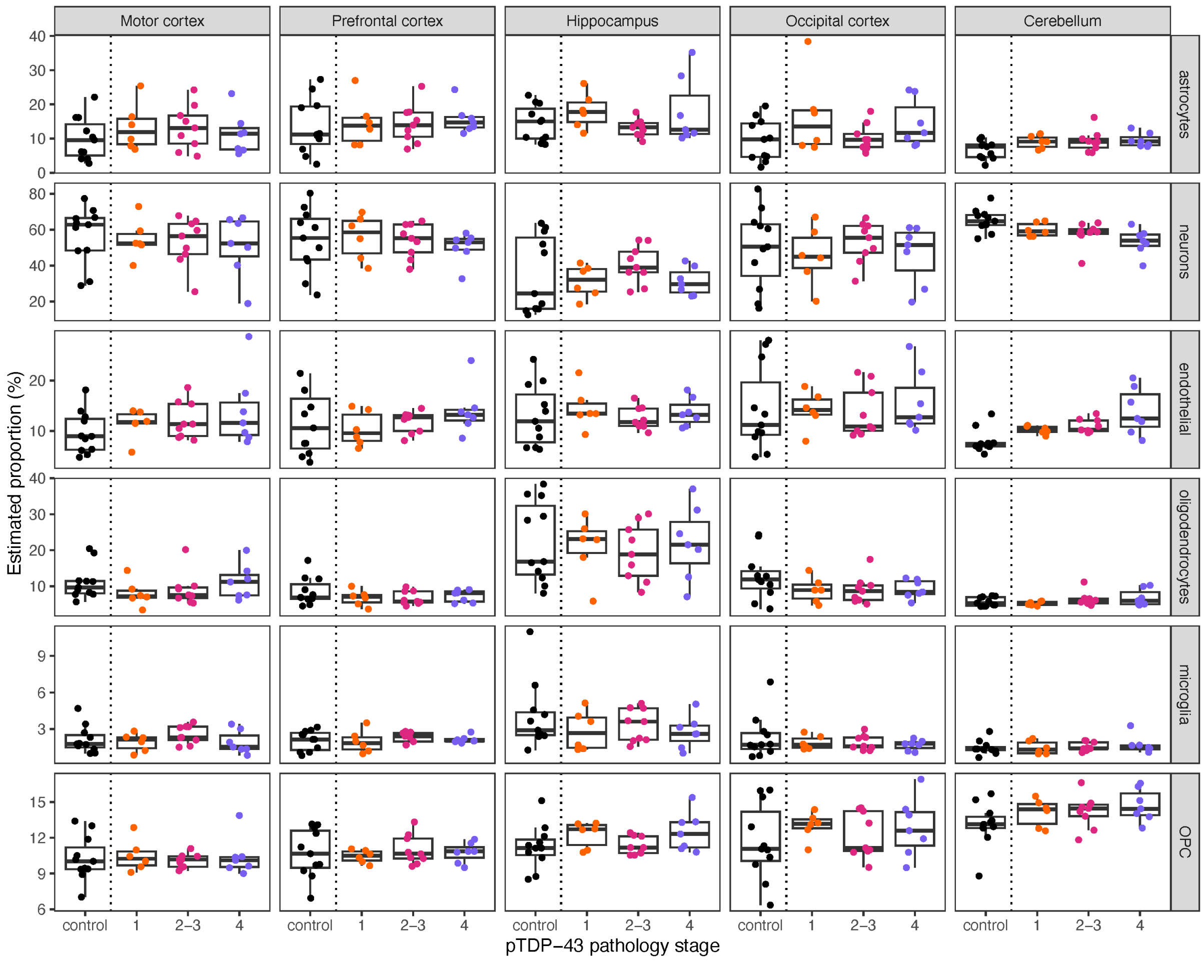

Fig. S20: Estimated proportions of six major cell types in ALS patient pTDP-43 pathology patient subgroups across all five brain regions. Cell-type deconvolution was performed using dtangle on all 165 brain RNA-seq samples. Here, cortex-derived single-nucleus RNA-seq (Darmanis et al., 2015) was used as the reference data. No cell type proportions were significantly different between ALS pTDP-43 pathology stage groups (Kruskal-Wallis rank sum test; significance threshold of p-value < 0.05). Controls were not included in statistical testing and are included here purely as a reference. OPC, oligodendrocyte progenitor cell.

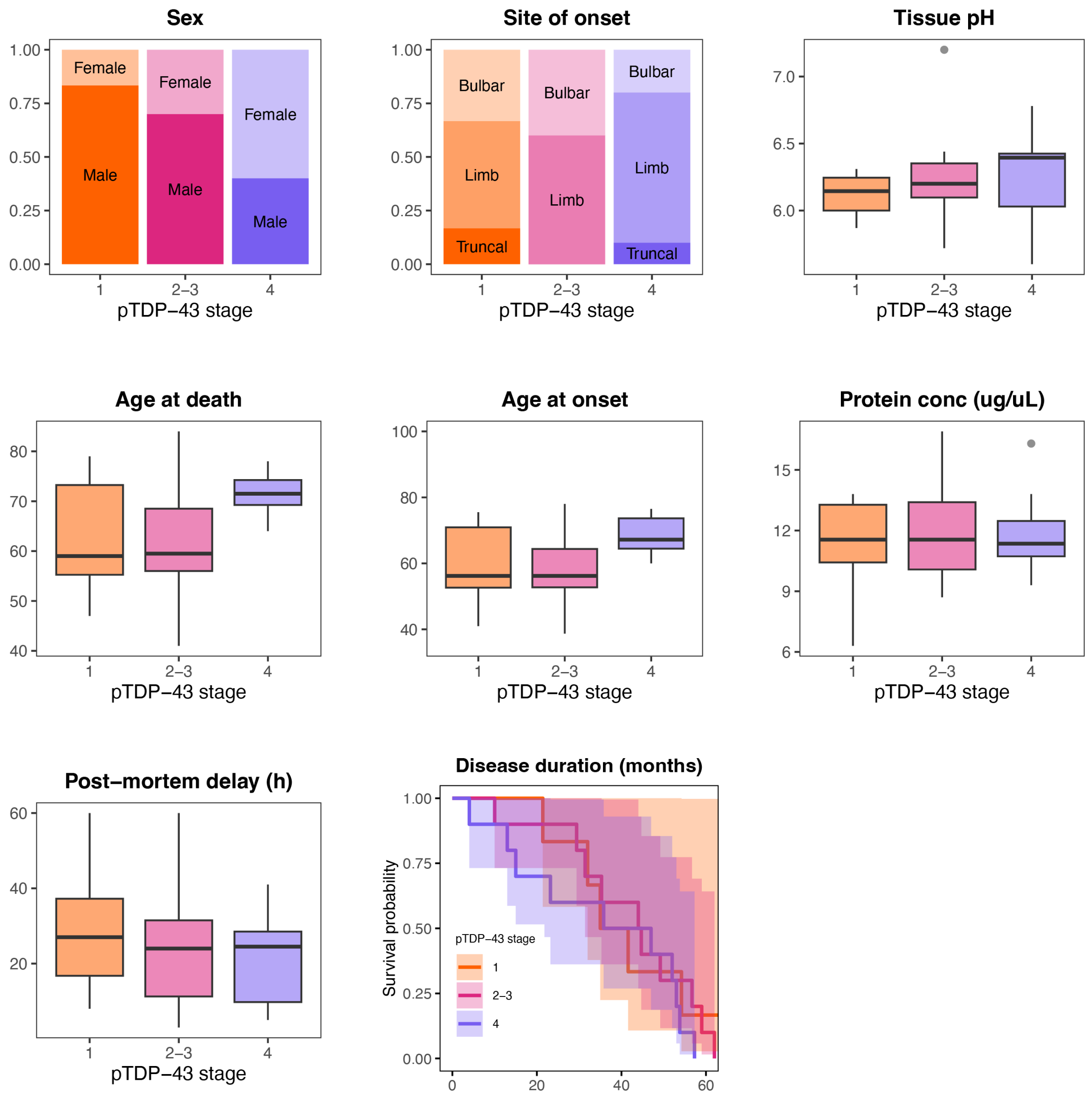

Fig. S21: Comparison of samples features between ALS patient pTDP-43 pathology stage groups (SWATH-MS cohort). ALS patients were were sub-categorised by their pTDP-43 pathology stage (6 stage 1, 10 stage 2-3, 10 stage 4). No features were significantly different between pTDP-43 pathology stage groups. Complete results of statistical comparisons can be found in Table S15.

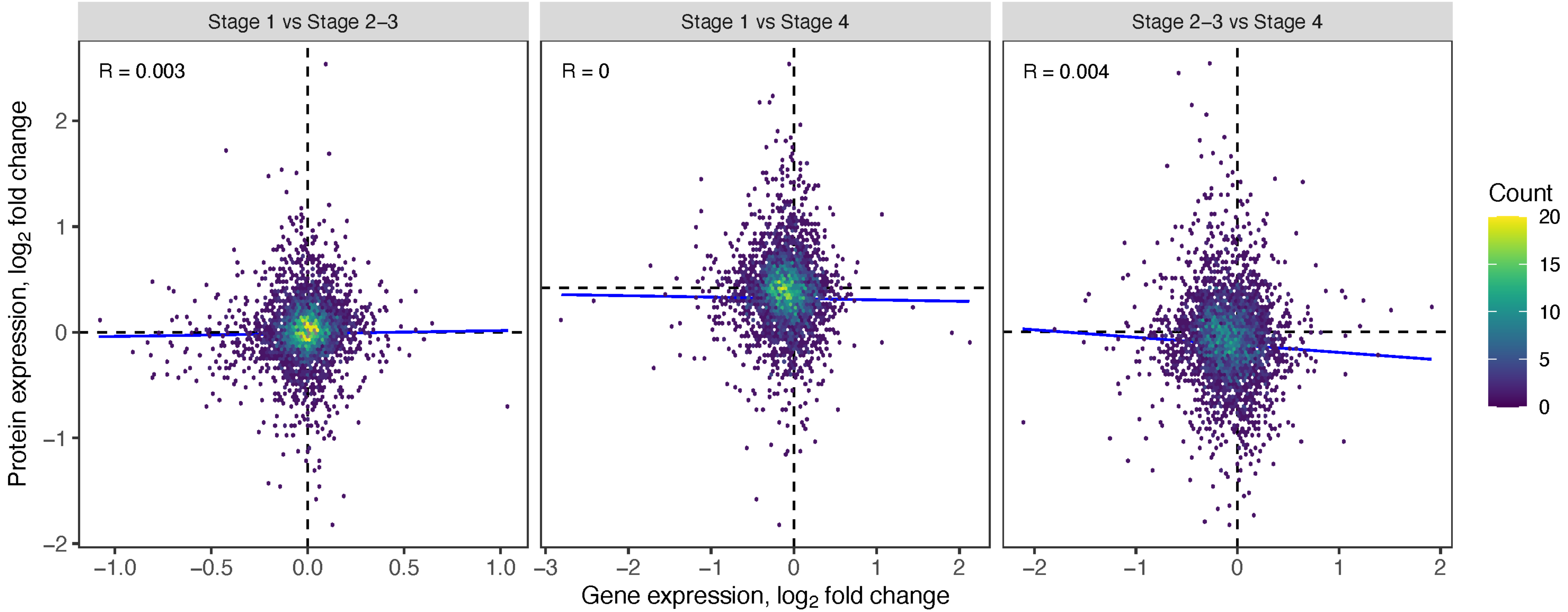

Fig. S22: Correlation of cerebellum gene and protein log_2 fold changes for each pTDP-43 pathology stage pairwise comparison. Each dot represents a gene/protein that was detected in both the RNA-seq and SWATH-MS analyses. Regression line is shown in blue with corresponding adjusted R^2 value shown at the top of each plot.

Supplementary Methods

Cohort selection

Post-mortem spinal cord RNA was excluded from the present study. We previously reported that an organic-based RNA extraction method was required to isolate high-yield RNA from post-mortem spinal cord but not brain tissue (Grima et al., 2022). The use of different RNA extraction methodologies between brain and spinal cord tissue may confound inter-region comparisons, which were the focus of this study. Furthermore, we observed significantly higher RNA integrity (RIN) for ALS patient- versus control-derived spinal cord RNA, which may confound ALS-control comparisons.

Cell-type deconvolution

We note that the proportion of motor cortex neurons estimated by cell-type deconvolution was not significantly reduced in ALS patients relative to controls (Figure 2). The cortical layer 5 upper motor neurons that undergo degeneration in ALS, make up a small proportion of the total neuron population and would have expression profiles that are highly correlated with other glutamatergic neurons. Both factors are known to negatively impact cell-type deconvolution accuracy (Sutton et al., 2022). It was therefore not feasible to specifically examine further motor cortex neuronal sub-populations using our existing data. We note that a previous publication performing cell-type deconvolution on bulk RNA-seq of ALS post-mortem motor cortex similarly did not observe a significant decrease in estimated neuron proportions relative to controls (Dols-Icardo et al., 2020).

Alternative splicing analysis

LeafCutter Docker container

To assist in the implementation of LeafCutter (Li et al., 2017), a Docker container was set-up. LeafCutter can be executed in Docker using `docker run mqmnd/leafcutter:amd-mamba2`. It is AMD compatible, and not tested on ARM. Note this software container is provided "as is" and is intended solely for research, educational, and informational purposes. It is not designed, developed, or tested for clinical use or for deployment in any environment that requires reliable performance under medical conditions.

Proteomics analysis of post-mortem cerebellum

Protein sample and local ion library preparation

Post-mortem cerebellum tissue from an extended cohort of 26 sporadic ALS patients (6 stage 1, 10 stage 2-3, 10 stage 4) and 14 controls were used for sequential window acquisition of all theoretical mass spectra (SWATH-MS) analysis. For each sample, 50-100 mg of fresh frozen tissue was homogenised in a solution of 1% sodium deoxycholate, 100 mM triethyl ammonium bicarbonate. Samples were homogenised at room temperature using a Precellys tissue homogeniser at 6,500 rpm for 20 seconds repeated three times before centrifuging at 15,000xg at 4°C for 10 min and collecting the supernatant. 50 μg protein aliquots were reduced with 10 mM dithiothreitol at 60°C for 30 min, alkylated with 25 mM iodoacetamide at room temperature in the dark for 30

min and digested with trypsin (1:55) for 16 h at 37°C. Sodium deoxycholate was removed by trifluoroacetic acid precipitation followed by desalting using Empore 47 mm SDB-RPS StageTips as previously described (Mirzaei et al., 2017). Samples were reconstituted in 50 μL 0.1% formic acid for liquid chromatography mass spectrometry (LC-MS).

To generate the local ion library, a pool of all 40 samples was prepared and fractionated by high pH reverse phase high performance liquid chromatography (RP-HPLC) using an Agilent 1260 HPLC coupled to a quaternary pump. The pooled sample was dried and resuspended in 6 mM ammonium hydroxide solution (pH 10.5; mobile phase A) before injection. 6 mM ammonia solution with 90% acetonitrile (pH 10.5; mobile phase B) was added at a flow rate of 300 μL/min before increasing the concentration from 3% to 30% over 55 min, then to 70% over 10 min and then to 90% over 5 min. The eluent was collected at 2 min intervals at the beginning of the gradient and at 1 min intervals for the rest of the gradient.

LC-MS acquisition

Peptides were separated and analysed on a TripleTOF 6600 mass spectrometer (SCIEX, MA, USA) coupled to an Eksigent NanoLC-Ultra HPLC system (Eksigent, CA, USA). 10 μL peptide samples were injected onto a peptide trap (5 mm × 300 μm, μ-Precolumn C18 PepMep 100 5 μm; Thermo Fisher Scientific, MA, USA) at 15 μL/min for 3 min before the gradient was switched in-line with an in-house packed column (20 cm x 75 μm, Reprosil-Pur 120 C18-AQ 3 μm; Dr Maisch, Germany). Peptides were eluted from the column using a linear solvent gradient with mobile phase A consisting of 0.1% formic acid and mobile phase B consisting of 99.9% acetonitrile, 0.1% formic acid with a flow rate of 300 nL/min. The concentration of mobile phase B was increased from 5% to 35% over 120 min for peptide elution. The column was cleaned with 95% mobile phase B for 6 min and then equilibrated with 5% mobile phase B for 10 min before next sample injection. Pooled sample LC eluent was subjected to positive ion nano-flow electrospray analysis in information dependent acquisition (IDA) mode. In IDA mode, a TOF-MS survey scan was acquired at m/z 350-1500 with 0.25 sec accumulation time, with the 20 most intense multiply charged ions (2^+ - 5^+; >200 counts/sec) in the survey scan sequentially subjected to MS/MS analysis. MS/MS spectra were accumulated for 100 msec in the mass range m/z 100-1800 with rolling collision energy. In SWATH mode, the same chromatography conditions were used, and individual sample LC eluents were subjected to positive ion nano-flow electrospray analysis in an independent acquisition mode. A TOF-MS survey scan was acquired (m/z 350-1500, 50 msec) then the 100 predefined m/z ranges were sequentially subjected to MS/MS analysis. MS/MS spectra were accumulated for 30 msec in the mass range m/z 350-1500.

Proteomics data processing and differential protein expression analysis

IDA-MS data was searched with ProteinPilot v5.0 (SCIEX) using the Paragon algorithm in thorough mode. The UniProt Homo sapiens reviewed proteome (20,375 proteins, downloaded April 2022) was used for searching the data, with carbamidomethylation of Cys residues selected as a fixed modification. An unused score cut-off of 1.3 (95% confidence for identification) and global protein FDR of 0.01 was used. PeakView v2.2 (SCIEX) was then used to extract SWATH-MS peak areas with the local ion library ($\sim$3,500 proteins) as reference. The top 6 most intense fragments of each peptide were extracted (75 ppm mass tolerance, 10 min retention time window). Shared and modified peptides were excluded. Peptides (max. 100 peptides per protein) with confidence $\geq$ 99% and FDR $\leq$ 0.01, based on chromatographic feature after fragment extraction, were used for quantitation. The ion peak areas exported by PeakView for each sample were normalized by total area normalisation as previously described (Wu et al., 2016). Dif-

ferential expression was assessed by fitting a linear model for the log transformed normalised protein peak areas with pTDP-43 pathology stage, sex, and age used as predictor variables (i.e. protein expression $\sim$ pTDP-43 stage + sex + age). Fold changes were calculated by taking the mean of the log-transformed areas of sample replicates and back-transforming before calculating the ratio between two conditions. Significant differentially expressed proteins were classified as those with a p-value <0.05 and fold change <0.67 or >1.5. To identify proteins defining each pTDP-43 pathology stage, direct comparisons were made between each subgroup. The overlap of proteins identified as significantly up- or downregulated from each comparison was classified as subgroup-defining genes.
